# Essential Oils as Green Antibacterial Modifiers of Polymeric Materials

**DOI:** 10.3390/polym17212924

**Published:** 2025-10-31

**Authors:** Kamila Majewska-Smolarek, Anna Kowalewska

**Affiliations:** Department of Polymeric Nano-Materials, Centre of Molecular and Macromolecular Studies, Polish Academy of Sciences, Sienkiewicza 112, 90-363 Łódź, Poland; kamila.majewska-smolarek@cbmm.lodz.pl

**Keywords:** antimicrobial polymers, essential oils, nanoencapsulation, nanofibers, nanocomposites

## Abstract

The need for new strategies to reduce the susceptibility of polymeric materials to bacterial colonization is growing, especially with the emergence of drug-resistant bacterial strains. Antimicrobial agents used to modify polymers should not only be effective against microorganisms in both planktonic and biofilm states but also be safe and environmentally friendly. Phytochemicals, which are components of essential oils, may be a suitable choice to help combat microbial resistance to antibiotics. Furthermore, they meet the requirements of green chemistry. Essential oils synthesized by plants as secondary metabolites are capable of combating both Gram-positive and Gram-negative bacteria by disrupting lipid bilayers, affecting efflux pumps, compromising the integrity of bacterial cell membranes, and inhibiting the quorum-sensing system. They are also effective as adjuvants in antibiotic therapies. In this review, we outline the mechanism of action of various essential oil components that resulted in enhanced eradication of planktonic bacteria and biofilms. We summarize the use of these antimicrobial agents in macromolecular systems (nanovessels, fibers, nanocomposites, and blends) and provide an overview of the relationship between the chemical structure of phytochemicals and their antimicrobial activity, as well as their influence on the properties of polymeric systems, with a special focus on green active packaging materials.

## 1. Introduction

**Figure 1:**
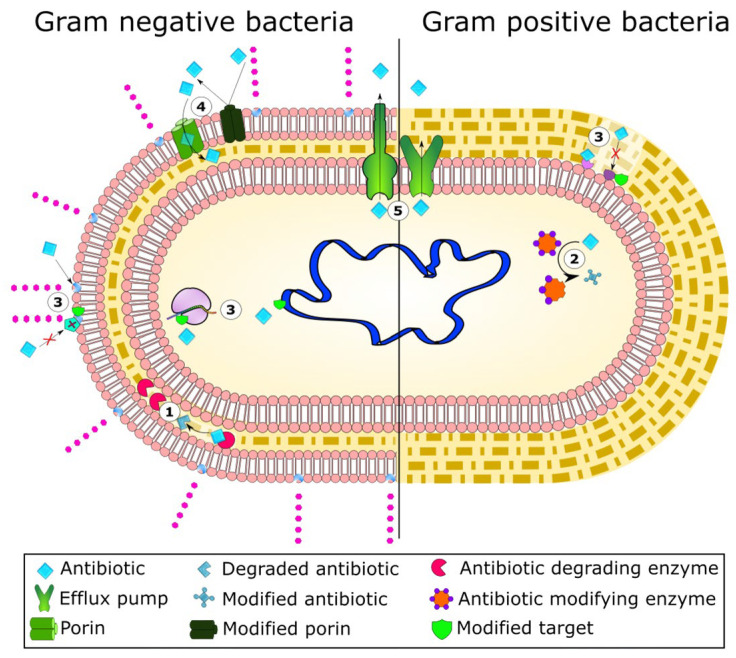
Mechanisms of antibiotic resistance in Gram- and Gram+ bacteria: (1) enzymatic hydrolysis; (2) enzymatic chemical modification; (3) modification of cell morphology (antibiotic targets); (4) modification of porin proteins; (5) extrusion of antibiotics by membrane efflux pumps [2].

Polymeric materials are commonly used in many fields, including biomedical applications, and the demand for new strategies to make them less prone to bacterial settlement is increasing. The increase in bacterial resistance to antibiotics and the emergence of drug-resistant strains of bacteria pose a huge threat, especially in the case of nosocomial infections. A wide range of conventional antimicrobial agents that induce bacterial cell death or suppress bacterial cellular activity and growth is currently available. However, the microbial pathogens tend to develop the ability to defeat their action. The mechanisms of this acquired resistance are based on changes of the cellular wall permeability to inhibit the entrance of antibiotics into the microbial cells, inactivation of the antibiotics, or their molecular targets inside the cells (Figure 1) [1,2].

The antimicrobial agents used for polymer modification should not only be effective against microorganisms in both planktonic and biofilm states but also safe and environmentally friendly. Some widely used substances with antibacterial properties, such as silver nanoparticles and quaternary ammonium compounds, often raise concerns about their potential toxicity to living organisms. Phytochemicals, which are the components of essential oils (EOs), can be the right choice as agents helping to combat the resistance of microorganisms to antibiotics [3]. Synthesized as secondary metabolites by plants of different species, they are capable of eradicating both Gram-positive (G+) and Gram-negative (G-) bacteria [4] and meet the requirements of green chemistry principles. Essential oils are complex mixtures of phenols, terpenes and terpenoids, alkaloids, saponins, and peptides, whose relative content can depend on the cultivation and harvesting conditions. Although their positive medicinal effect is well known, the precise mechanism of action is still the subject of ongoing studies. It has been established that EOs can impair the integrity of bacterial cell membranes and alter their permeability. Significant therapeutic effects have been also achieved when combining plant-derived compounds with conventional antibiotics. The phytochemicals appear to be interesting candidates also for the development of new-generation antibacterial polymeric materials. The inclusion of EOs in macromolecular matrices can be an effective and safe strategy to extend the time of their interaction with pathogens [5,6,7]. Although remarkable advances have been made in this field, their application can encounter practical problems due to their volatility and low solubility in water. One should be also aware of the adversary health effects of some essential oils (e.g., allergic reactions) when applied in high concentration.

Given the significant value of various phytochemicals in the design of environmentally friendly yet effective antibacterial and anti-adhesive polymers, including coatings, fibres, textiles, membranes, packaging materials, etc., and in order to fully exploit the potential of these natural substances, it is particularly important to understand the complex mechanism of action of essential oils on bacteria and the effects of their chemical modification (including covalent attachment to the polymer chain) or the use of a specific macromolecular system to improve the bioavailability and stability of essential oils.

In this review, we present the influence of the chemical structure of the main components of essential oils (monoterpenoid phenols, acyclic terpenes, and linear monoterpenoids) on their antibacterial and antibiofilm efficacy in the context of applications in polymeric materials chemistry. To elucidate the mechanism of action of various essential oil components (eugenol, carvacrol, linalool, geraniol, citronellol, farnesol, and phytol) against Gram-positive and Gram-negative bacteria, we briefly characterized the phenomenon of quorum sensing and its role in bacterial biofilm formation. We also described the interactions of phytochemicals with microorganisms with different cell wall morphologies; their interference with lipid bilayers; and their role in influencing the mechanism of efflux pumps, cell wall disruption, and inhibition of the quorum-sensing system. There are numerous examples in the literature of supramolecular interactions between phytochemicals and bacterial proteins. This information, obtained through *in silico* docking, is essential for both the design of anti-adhesive polymer systems and understanding their antibacterial activity. In the second part of the review, we also revised methods for incorporating plant-derived antimicrobials into various polymeric materials, including nanoscale formulations (nanocapsules, nanofibers, and inclusion complexes) and larger-scale applications in nanocomposites and blends (prepared by physical mixing and covalent grafting), including active packaging materials based on synthetic and natural biopolymers. We hope that this review will contribute to a better understanding of the action of phytochemicals, as well as inspire and enable their even more effective use in the design of modern macromolecular antibacterial systems for various applications.

## 2. Interactions of Essential Oils with Gram-Positive and Gram-Negative Bacteria

### 2.1. Bacterial Biofilm and Quorum Signaling

**Figure 2:**
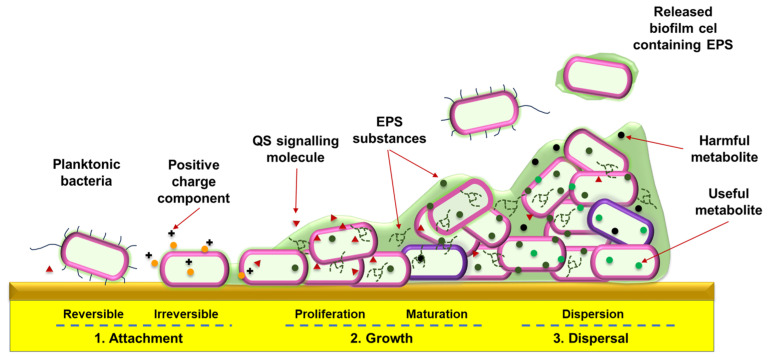
Schematic representation of the three major stages of biofilm formation, growth, and maturation [8]. Reprinted from Infect Genet Evol., 112, Hajiagha, M.N.; Kafil, H.S., Efflux pumps and microbial biofilm formation, 105459, Copyright (2023), with permission from Elsevier.

The main survival strategy of microorganisms is the formation of biofilms—complex colonies with a three-dimensional structure in which cells attached to a surface are isolated/protected from the external environment by a polysaccharide envelope. This makes biofilm bacteria much more resistant to antibiotic treatment than the planktonic phenotype. In early stages of biofilm formation (Figure 2), bacteria attach to surfaces (biotic or abiotic) using adhesive factors. This stage is followed by proliferation of the adhered bacteria and the formation of a multifunctional biofilm matrix composed of extracellular polymeric substances (EPS) that surrounds the cells (biofilm maturation). In the final phase, planktonic cells are released from the mature biofilm to form new communities.

The mature biofilm architecture contains channels and pores for intercellular communication within the microcolony, circulation of nutrients and toxic products, and adaptation to changes in environmental conditions (e.g., changes in pH and osmolarity). The increased tolerance of bacterial biofilms to conventional antibiotics has prompted extensive research into the identification of natural substances that could serve as alternatives to antibiotics, and their application in materials chemistry and engineering [9,10].

**Figure 3:**
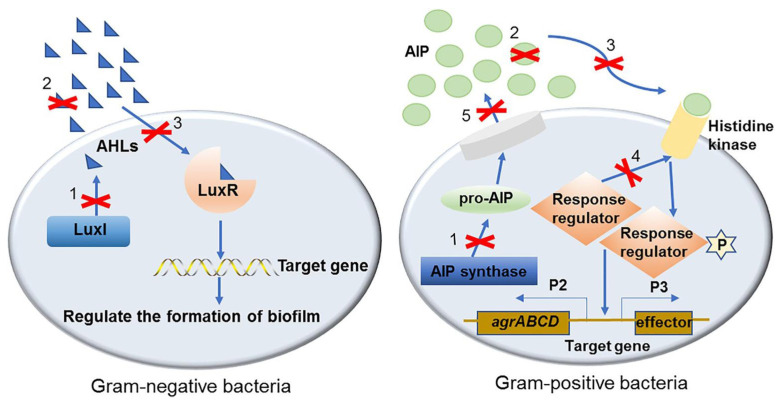
Modes of action of QS inhibitors to control biofilms formed by G- and G+ bacteria: (1) inhibition of AI synthesis; (2) degradation or inactivation of AIs by specific enzymes (AHL lactonase and/or oxidoreductase); (3) inhibition of signal receptors by AI antagonists; (4) disruption of the signal cascade by interference with response regulators; (5) suppression of AI efflux by reducing their extracellular levels. Adapted from Zhou et al. [12] Regulatory Mechanisms and Promising Applications of Quorum Sensing-Inhibiting Agents in Control of Bacterial Biofilm Formation. Front. Microbiol. 11, 589640. https://doi.org/10.3389/fmicb.2020.589640. Copyright © 2020 Zhou, Zhang, Ge, Zhu and Pan. This is an open-access article distributed under the terms of the Creative Commons Attribution License (CC BY).

There are several possible strategies for combating biofilm-related infections. These include treatment with substances capable of destroying the biofilm matrix or resistant cells, or the use of agents that enhance the action of antimicrobials. Unfortunately, antibiotic treatment quite often results only in the partial killing of bacteria, allowing the surviving cells to spread the infection. Biofilms can also produce specific enzymes that deactivate antibiotics. Coordinated cooperation between individual cells within the biofilm is crucial for the functioning of the microbial community [11]. This requires sophisticated signalling mechanisms: the expression of molecular messengers (“autoinducers”, AIs), their release into the extracellular environment, followed by recognition by specific receptor proteins and transduction leading to a coordinated response. This process of communication between G+ and G- bacteria is called quorum sensing (QS) and allows microorganisms to adapt effectively to their environment. Owing to this microbial density-dependent phenomenon, pathogens can measure their population and modulate the expression of genes responsible for the secretion of virulence factors, competence, and biofilm formation. Bacteria exhibit two main QS mechanisms, which involve separate signalling pathways (Figure 3) [12].

QS in G- bacteria (e.g., *Escherichia coli*, *Pseudomonas aeruginosa*, *Aeromonas hydrophila*, *Legionella*, *Klebsiella pneumoniae*, *Helicobacter pylori*, and *Salmonella*) is typically based on the synthesis of acylated homoserine lactones (AHLs). AHLs can transfer the bacterial membrane into the extracellular environment and enter the cell, where they meet specific receptors (LuxR) and form an AHL-LuxR complex that binds to bacterial DNA, thereby regulating the transcription of specific genes at concentrations above the required AHL threshold. The receptor is also a transcription factor, and the formation of the AHL-LuxR complex catalyses AHL synthesis. This quickly leads to a synchronous response of the entire microorganism population. In G+ bacteria (e.g., *Staphylococcus aureus*, *Streptococcus pneumoniae*, *Listeria monocytogenes*, and *Clostridium difficile*), autoinducing peptides (AIPs), which consist of several (5–17) amino acid units, act as signalling molecules. The transfer of signal peptides to the extracellular environment requires special transporters, which are detected by a histidine kinase sensor and cause signal transduction through the phosphorylation of intracellular transcription factors [13]. Some G+ bacteria can use ɣ-butyrolactones [14] or α-pyrone [15] as QS molecules. Both G+ and G- bacteria can produce a special autoinducer (AI-2—cyclic furanosyl borane diester), which can serve as a “universal” quorum signal for interspecies communication [16].

The use of quorum-sensing inhibitory molecules, which can disrupt this phenomenon (quorum quenching (QQ) by interfering with and capturing QS factors synthesized by bacteria), appears to be an interesting possibility for combating harmful microorganisms and reducing their tendency to form biofilms. This approach, based on mimicking and disrupting bacterial communication, is all the more important as young biofilms are easier to eliminate. Research is ongoing into new therapeutic substances that could act as antibacterial (but non-toxic) agents without promoting the development of acquired resistance in bacterial strains [17]. Among the many chemicals that can act as QQ agents, essential oils (EOs) occupy an important place as they can effectively prevent biofilm formation and destroy the already existing biofilm. It has also been postulated that EOs can disrupt microbial quorum communication systems by acting on factors that regulate microbial activity [18,19,20]. The components of essential oils were shown to be more effective than conventional antibiotics in inhibiting biofilm formation and may be used as a substitute for antibiotics. The single components of essential oils or their mixtures can be combined with other antibiotic substances to decrease the effectiveness of QS [3,21].

### 2.2. The Influence of the Cell Wall Structure of G+ and G- Bacteria on Their Interactions with Essential Oils

**Figure 4:**
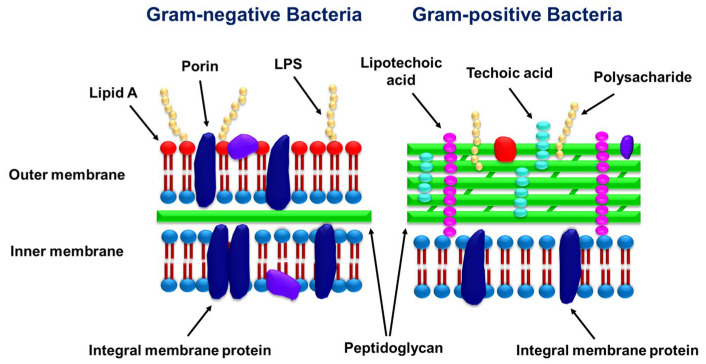
Schematic structure of the cell walls of G-positive and G-negative bacteria. Adapted from Huan et al. [22] Antimicrobial Peptides: Classification, Design, Application and Research Progress in Multiple Fields. Front. Microbiol. 11, 582779. https://doi.org/10.3389/fmicb.2020.582779. Copyright © 2020 Huan, Kong, Mou and Yi. This is an open-access article distributed under the terms of the Creative Commons Attribution License (CC BY).

Bacterial quorum sensing can be inhibited by various mechanisms, which depend on the chemical composition of essential oils and their concentration. However, a crucial factor is the morphology of the bacteria cell walls (Figure 4) [3,18,22].

The mechanism by which components of essential oils affect G+ cells is well established—these microorganisms are generally less resistant to plant-based chemicals than G- bacteria due to the fact that their cell walls contain a thick layer of peptidoglycan [18,23]. Essential oils are lipophilic and can penetrate the membrane to remain between phospholipids and/or affect membrane lipid synthesis. This leads to alterations in cell membrane permeability and degradation of the cell wall, cytoplasmic membrane, and membrane proteins.

G- bacteria are less susceptible to the action of chemicals due to the presence of an outer double phospholipid membrane, connected to a thin layer of peptidoglycan by lipopolysaccharides. Penetration of such a complex barrier is difficult, and antibiotic molecules can only enter the cell (selectively) through porins (proteins that form water-filled channels in the cell wall) [24]. Essential oils can act against G- bacteria antagonistically by inhibiting AHL synthesis and blocking its transport and/or secretion, or by capturing AHL molecules outside the bacterial cell, thus inhibiting their binding to the AHL receptors in other cells. For example, clove essential oil (main component: eugenol) tested on G- strains of *P. aeruginosa* and *A. hydrophila* at concentrations below the inhibitory threshold significantly reduced virulence factors regulated by las and rhl (LasB, total protease, swimming motility, chitinase, pyocyanin, and exopolysaccharide production) and decreased their ability to form biofilms [25].

**Figure 5:**
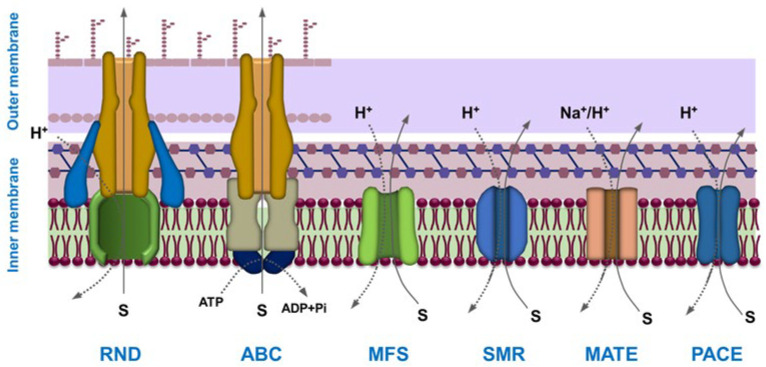
Schematic representation of different types of bacterial transporters located in the cell membrane of G+ and G- bacteria (major facilitator superfamily (MFS), ATP-binding cassette (ABC), small multidrug resistance (SMR), multidrug and toxin extrusion (MATE)] and exclusively in G- species [resistance nodulation-cell division (RND)) (OM, outer membrane; IM, inner membrane; S, substrate) [8]. Reprinted from Infect Genet Evol., 112, Hajiagha, M.N.; Kafil, H.S., Efflux pumps and microbial biofilm formation, 105459, Copyright (2023), with permission from Elsevier.

There are also structural features that characterize both G+ and G- bacteria: efflux pumps (EP). EP are membrane proteins that not only remove toxins and metabolites from the cell but also excrete quorum-sensing mediators and EPS molecules into the environment (Figure 5) [26,27]. Unlike G- bacteria, whose cell wall structure allows for easier removal of substances, G+ bacteria have more diverse efflux pump systems.

EPs are a main factor in bacterial resistance to antimicrobial agents [28] and biofilm formation [8]. Recent studies explained the role of phytochemicals in inhibiting the action of efflux pumps in cell membranes. Terpenes and terpenoids, which are main components of essential oils, can act as inhibitors of bacterial efflux pumps and enhance the action of antibiotics against resistant G+ and G- bacteria (e.g., NorA efflux pump in *S. aureus*, AcrAB-TolC in *E. coli*, and MexAB-OprM in *P. aeruginosa*), with a significantly higher effectiveness in G+ strains [29,30]. Inhibition of efflux pumps by essential oils underlies the synergistic mechanism of action of phytochemicals in combination with beta-lactam antibiotics [31].

## 3. Antibacterial and Antibiofilm Activity of the Main Components of Essential Oils—The Influence of the Chemical Structure on Their Metabolism and Effectiveness in Quorum Quenching

**Figure 6:**
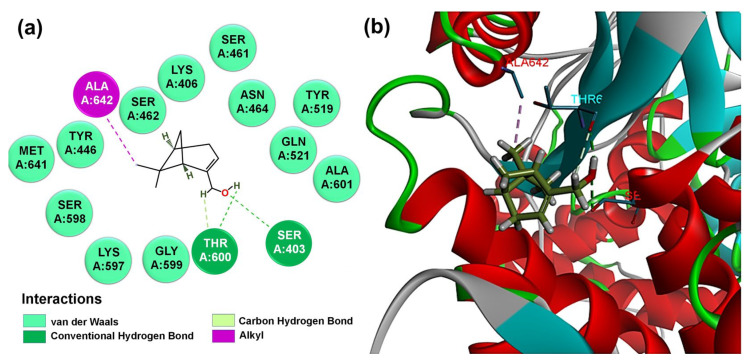
An exemplary molecular docking analysis: (**a**) two-dimensional and (**b**) three-dimensional representation of interactions between a phytocomponent and a protein active site (as an example, the complex of myrtenol and the active sites of PBP2) [37].

Components of essential oils (including carvacrol, carvacrol methyl ester, thymol, eugenol, geraniol, geranyl acetate, R(+)-limonene, (2)-linalool, and *cis*/*trans* citral) can be bactericidal or bacteriostatic, depending on the concentration used. The antibacterial properties of these compounds are related to their chemical structure and physicochemical properties (lipophilicity, partition coefficient, and type of hydrogen bonding) [31,32]. Key factors that increase the antibacterial activity of EOs by improving their interactions with bacterial cell membranes are the presence of specific reactive groups (phenolic hydroxyl or aldehyde carbonyl), long alkyl chains, and unsaturated bonds. *In silico* docking by molecular modelling interactions between phytochemicals and amino acids in proteins at active site pockets can provide information on the observed biological activity [33,34,35,36]. Such a structural analysis helps predict the main binding sites in the complexes and estimate the energies of binding, which are influenced by the type of supramolecular interactions (hydrogen bonds, electrostatic, or π-π interactions) (e.g., Figure 6).

### 3.1. Monoterpenoid Phenols

**Scheme 1:**
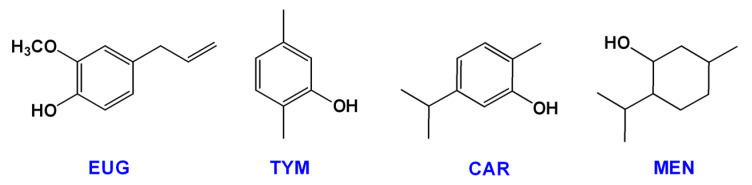
Chemical structures of antimicrobial monoterpenoid phenols: eugenol (EUG), thymol (TYM), carvacrol (CAR), and menthol (MEN).

The activity of phenolic monoterpenes (e.g., carvacrol, eugenol, thymol, and menthol) (Scheme 1) can be mainly attributed to the presence of a hydroxyl group at the aromatic or cyclohexane ring [38]. The mechanism of their action against G+ and G- bacteria indicates a relationship between structure and activity, depending on the length of the side hydrocarbon chain and the number and position of substituents at the ring core.

Studies on the antibacterial activity of eugenol, thymol, carvacrol, and their respective 2- and 4-allyl, 2-metallyl, and 2- and 4-n-propyl derivatives against planktonic and *S. epidermidis* and *P. aeruginosa* biofilms have shown that the allyl derivatives were more active in both killing and inhibiting planktonic cells but less effective against biofilms [39]. The effect was attributed to the slower penetration of the modified compounds into the biofilm and their different metabolism by cells in the biofilm.

Phenolic monoterpenes may also affect microbial quorum sensing. For example, it was shown that thymol (2-isopropyl-5-methylphenol) can block QS among various G- bacteria by affecting AHL production and suppressing flagellar gene transcription, thereby reducing bacterial motility and biofilm formation. *P. aeruginosa* and *A. hydrophila* were sensitive to peppermint oil (main component: menthol (5-methyl-2-(propan-2-yl)cyclohexan-1-ol)) at sub-minimum inhibitory concentrations [40]. The production of AHL-regulated virulence factors and biofilm formation were significantly impaired in this case by the action of menthol on the Las and Pqs QS systems. Phenolic monoterpenes can also sensitize multidrug-resistant strains to bactericidal or bacteriostatic antibiotics [41]. Eugenol and carvacol were selected to show the antibacterial potential of monoterpenoid phenols in more detail in this review.

#### 3.1.1. Eugenol

Eugenol [2-methoxy-4-(prop-2-en-1-yl)phenol] (EUG) can be found in essential oil extracts of plants such as cloves, cinnamon, bay leaf, or nutmeg. It is well known for its antiseptic, antioxidant, anticancer, anesthetic, and repellent/insecticidal properties [42]. The presence of three chemically active sites in the EUG structure (hydroxyl, allyl, and aromatic) makes it an effective antimicrobial agent and an important precursor in the synthesis of natural products and new drugs [42]. EUG acts on the cytoplasmic membrane in both G+ and G- bacteria. The minimum inhibitory concentration (MIC) and minimum bactericidal concentration (MBC) of EUG are usually low (for example, against E. coli, MIC = 0.125 μg/mL; MBC = 0.250 μg/mL [43]). The ability of EUG to scavenge reactive oxygen species may be also attributed to the phenolic structure. Masking the critical functional groups can reduce the effectiveness of EUG, although some targeted structural modifications were shown to increase its antibacterial activity [38]. For example, acetylation of eugenol decreased its activity against E. coli and made it inactive against S. aureus, whereas epoxidation enhanced its activity against the latter [44]. Esterification of the EUG hydroxy group with various carboxylic acids and addition reactions at the double bond of the allyl group increased its antimicrobial efficacy against E. coli and S. aureus [45]. Acylation and alkylation of EUG made it bacteriostatic against selected G+ and G- strains but bactericidal only against G+ strains [46]. The presence of an NO_2_ group in the para-position increased the specificity against G+ strains, with no activity against G- strains at concentrations up to 500 µg mL^−1^. This information is of high significance in the context of the use of EUG (and other phytophenols) for the covalent modification of polymeric materials.

*In silico* docking has shown the importance of all structural features of EUG molecules in supramolecular binding with bacterial proteins. Eugenol can bind via hydrogen bonds to catalytic and other key amino acid residues of extended-spectrum beta-lactamase (ESBL) proteins produced by bacteria (*E. coli* and *K. pneumoniae*) [47]. Below the inhibitory level, it was shown that EUG can affect QS (lasB and pqsA) in *E. coli* by limiting the production of virulence factors and biofilm formation [48]. EUG also affected the virulence of *S. aureus* by interfering exotoxin generation through the repression of agrA transcription [49], blocking staphyloxanthin production by affecting *crtM* expression and CrtM (4,4’-diapophytoene synthase) enzymatic activity [50], binding to enoyl-[acyl-carrier-protein] reductase [NADPH] FabI through multiple pathways, and activating the oxidative stress-mediated apoptosis pathway of *S. aureus* [51]. Molecular docking also showed favourable interactions between EUG and the *S. aureus* NorA efflux pumps through hydrogen bonds and hydrophobic interactions [52], as well as MexA and AcrA efflux pumps in *P. aeruginosa* and in *E. coli* [53]. It was also demonstrated that eugenol and linalool can reduce the genomic DNA and RNA content and complex quorum-sensing proteins of *S. aureus* [54]. Molecular docking and 3D-binding orientation assessments proved that EUG can form a stable complex trough van der Waals interactions, hydrogen bonds, and π–alkyl interactions with hydrophobic residues of selected amino acids (proline, alanine, valine, glycine, and isoleucine) at the tip of csuE [55] in Csu pili, which is an adhesive organelle of *Acinetobacter baumannii*, crucial for their assembly and biofilm formation [56]. Molecular modelling also suggested that both EUG and cinnamaldehyde can interact with the receptor LasR protein in *P. aeruginosa* (binding energies of −6.09 and −5.72 kcal/mol for eugenol and cinnamaldehyde, respectively) [57]. The interactions involve intermolecular hydrogen bonds between the functional groups in eugenol and cinnamaldehyde and the amino acids of the LasR protein, as well as π-π stacking and π-π T-shaped and π–alkyl contacts.

#### 3.1.2. Carvacrol

Carvacrol (5-isopropyl-2-methyl phenol) (CAR) is one of the components of EOs that is most active against both G+ and G- bacteria. The mechanism of action of CAR is similar to other phenolic compounds (cell membrane damage, alteration of bacterial metabolism by inhibition of efflux pumps and membrane ATPases, inhibition of bacterial motility, and prevention of biofilm formation [58]). Carvacrol exhibited antimicrobial activity against KPC-producing *K. pneumoniae* [59] and antibiofilm and antivirulence activities against uropathogenic *E. coli* (UPEC) [60]. CAR reduced fimbriae production and the swarming motility of UPEC and markedly decreased its hemagglutinating ability. At sublethal concentrations, CAR also inhibited the formation of biofilms of *Chromobacterium violaceum*, *Salmonella enterica* subsp. *typhimurium*, and *S. aureus* but had a much weaker effect on *P. aeruginosa* biofilms [61]. The efficacy of CAR in those experiments was comparable with that of ciprofloxacin. Carvacrol can bind to quorum-sensing receptors in *P. aeruginosa* (RhlR, LasR, and PqsR), inhibiting/modulating biofilm formation and the production of certain virulence factors [62]. The activity of carvacrol against *C. violaceum* biofilm was also ascribed to the quorum-quenching effect [63]. CAR reduced the expression of gene coding for the N-acyl-l-homoserine lactone synthase and decreased the production of violacein and the activity of chitinase (both regulated by quorum sensing). Moreover, CAR can amplify the effect of tetracycline, erythromycin, and fluconazole [64].

### 3.2. Acyclic Terpenes and Monoterpenoid Alcohols

**Scheme 2:**
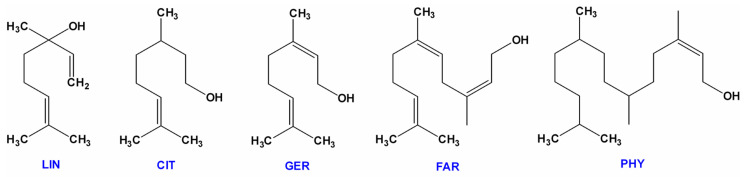
Chemical structures of antimicrobial acyclic terpenes (linalool (LIN)) and terpenoid alcohols (citronellol (CIT), geraniol (GER), farnesol (FAR), and phytol (PHY)).

**Figure 7:**
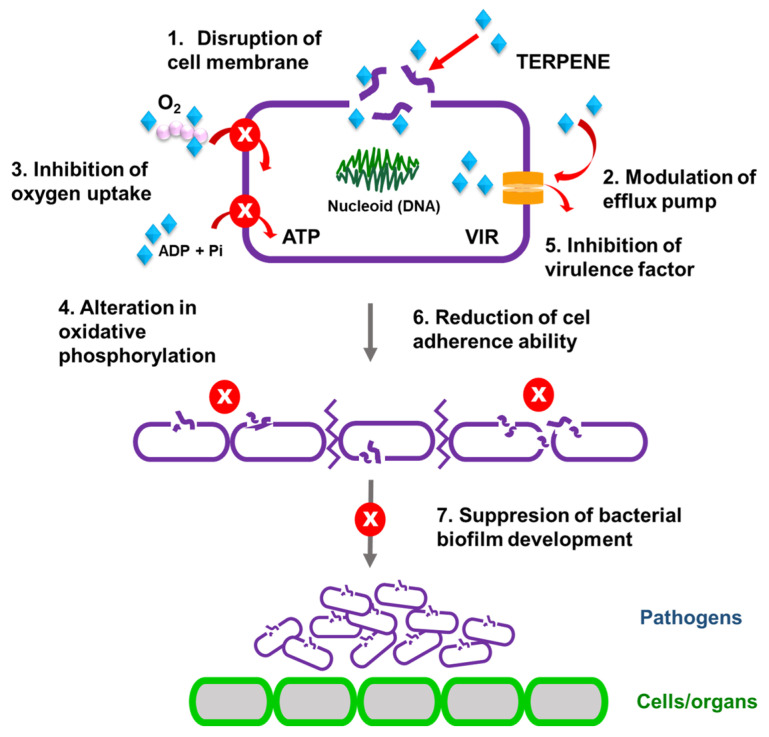
Schematic mode of action of terpenes on bacterial cells [66].

The chemical structure of linear terpenes and terpenoid alcohols (Scheme 2) facilitates their interactions with the lipophilic tails and polar groups of intermembrane lipids, thereby influencing lipoidal intermembrane and polar transmembrane pathways [65]. The postulated complex mechanism of their antimicrobial action and lipid membrane activity involves disrupting the cell membrane integrity, increasing the permeability of cell membranes by binding to lipids and other membrane components (efflux pumps), destructive interactions with important intracellular structures, the inhibition of virulence factors, and preventing biofilm formation by reducing the cells’ adherence ability (Figure 7). It was also shown that terpenes can hinder two crucial processes vital for the survival of microorganisms: oxygen uptake and oxidative phosphorylation (modification in cellular respiration) [66].

The antimicrobial activity of terpenes and terpenoids (e.g., carvone, limonene, and borneol) also depends on the content of active enantiomers (α-isomers were found to be significantly less active than β-isomers, and cis-isomers are inactive in contrast to trans-isomers) [67]. EOs mainly containing terpenes (such as *p*-cymene or limonene) are usually not very strong antimicrobial agents and are more effective against G- than G+ bacteria. Modelling the interactions *in silico* confirmed the role of the morphology of linear terpenes and terpenoids in their antibacterial activity. The presence of oxygen atoms or double bonds in the chemical structure enhances the inhibitory effect (mainly against G- bacteria). Monoterpenes with a carbonyl group, especially aldehydes, are known for their strong antimicrobial activity. An increase in the electronegativity of aldehydes (e.g., through the conjugation of an aldehyde group with a C=C bond) can further enhance the antibacterial effect. The following subsubsections review the antibacterial potential of selected linear terpenes and terpenoids.

#### 3.2.1. Linalool

Linalool (3,7-dimethylocta-1,6-dien-3-ol) (LIN) is an aliphatic terpene alcohol that has a complex effect on the G- and G+ cells. It is postulated that its antibacterial and antibiofilm action is based on cell membrane destruction, alterations of amino acid metabolism (a decrease in the cellular activity of alkaline phosphatase and an increase in the concentration of histidine and methionine, which regulate the structural phenotype of the bacterial biofilm, disturbing pH homeostasis by affecting the concentration of lysine and L-aspartate), and a change in metabolism of carbohydrates and lipids [68]. LIN inhibited the bacterial growth of *P. aeruginosa* [69] and *A. hydrophila* [70]. *In silico* docking revealed that the binding energies of LIN with the *P. aeruginosa* virulence factor regulator (PDB: 2OZ6) and *S. aureus* pyruvate carboxylase (PDB: 3HO8) were –5.6 and −4.7 kcal/mol, respectively (compared to −8.7 and −6.6 kcal/mol for a standard antibiotic drug ciprofloxacin) [71].

It was also shown that LIN can inhibit the growth of planktonic cells of G- *A. baumannii* and the formation of biofilm by hindering its quorum-sensing system [72] and displayed a strong bactericidal effect on clinical *E. coli* strains, regardless of their planktonic or biofilm form (lowering the *pgaABCD* gene expression and the formation of EPS) [73]. LIN and EUG inhibited the formation of *P. aeruginosa* biofilm, affecting the synthesis of quorum-sensing proteins (LasA and LasB) and virulence factors (pyocyanin and rhamnolipids) [74]. A reduction in protein and carbohydrate content in the extracellular polymeric substance was also observed. *In silico* modelling proved the effect by showing interactions between both EUG and LIN with the proteins involved in QS. Interestingly, linalool showed higher antimicrobial and antibiofilm activities against biofilm methicillin-resistant *S. aureus* (MRSA) and vancomycin-resistant *S. aureus* (VRSA) than carvacrol and eugenol [75]. The antimicrobial resistance patterns changed to sensitive phenotypes after treatment with EOs and methicillin or vancomycin. According to molecular docking results, linalool showed the highest and stable binding capacity (−6.00 Kcal/mol). The hydroxyl group of LIN was involved in H-bond formation with GLN506, whereas the terminal methyl group at position 8 of linalool could form H-π with TYR366. In the same comparative studies, it was shown that carvacrol formed a stable complex with Bap (binding energy of −5.59 Kcal/mol), and its phenyl moiety was engaged in a π-H bond with ARG738 (−5.44 Kcal/mol). Metabolomic analysis revealed that the action of LIN against methicillin-resistant *S. aureus* targeted the synthesis of core amino acids and glutathione pathways [76]. Molecular docking indicated a binding affinity between linalool and glutathione (−14.98 KJ/mol). Under treatment with LIN, some metabolic pathways were downregulated (biosynthesis of valine, leucine, and isoleucine, the pentose phosphate pathway, metabolism of 2-oxocarboxylic acid and D-alanine, insulin secretion), whereas the metabolism of heterocyclic compounds, organic acids, lipids and lipid-like molecules, phenylpropanoids, and polyketides was upregulated. The minimum biofilm inhibitory concentration of linalool against pellicle biofilms forming G+ *Bacillus amyloliquefaciens* DY1a and DY1b was 4 μL/mL and 8 μL/mL, leading to ~77% and ~83% biofilm eradication, respectively [77]. LIN also inhibited the motility of *B. amyloliquefaciens*, the production of EPS, and proteins of its biofilm matrix. Molecular docking analysis demonstrated, in this case, that LIN strongly complexed the quorum-sensing ComP receptor and biofilm matrix assembly TasA through intermolecular H-bonds, hydrophobic contacts, and van der Waals forces.

#### 3.2.2. Geraniol

Geraniol [(2*E*)-3,7-dimethylocta-2,6-dien-1-ol] (GER) is a lipophilic acyclic monoterpene, biosynthesised in, e.g., roses. Its antimicrobial activity and antioxidant and anti-inflammatory properties are well known [78,79]. GER was shown to be a promising antibiotic against *Streptococcus* spp. [80] and *Staphylococcus* spp. [81,82]. Its inhibitory effect against *S. aureus* (MRSA) was similar to that of vancomycin [83,84]. A comparison of the antimicrobial activity of geraniol and the compounds of its chemical transformation (such as citral (3,7-dimethylocta-2,6-dienal)) regarding selected G+ bacteria (the reference strains *S. aureus* ATCC 29213 and *Enterococcus faecalis* ATCC 29212 and the clinical strains *S. aureus* MRSA, *Staphylococcus epidermidis*, *E. faecalis* VRE VanB, *Enterococcus faecium* VRE VanA, and *E. faecium* VRE VanB) showed that citral was the most active against the *Staphylococcus* genus, followed by LIN and then GER [66]. Citral was also the most active against *Enterococcus* strains. However, in this case, GER and LIN were only slightly less active. A rapid bactericidal effect of geraniol and citronellol against *E. coli* [85] and *H. pylori* [86] was also noted.

Geraniol caused 85% inhibition of *S. epidermidis* RP62A biofilm [81] and a high quorum-quenching effect on a dual-species biofilm (*Erwinia carotovora* and *Pseudomonas fluorescens*) with an inhibition rate of > 60% [87]. The QQ effect was tentatively attributed to the inhibition of the AI-2 signal molecule. The combination of GER and hexanal also proved to be an efficient factor in quenching quorum of *P. fluorescens* [88]. Molecular docking analysis suggested that the inhibitory effect can be attributed to the action of hexanal, which fits into the minor groove of pcoI/pcoR DNA fragments. Both hexanal and geraniol showed high binding affinity with the PcoI protein (−31.49 kcal/mol and −25.72 kcal/mol, respectively) and thus could compete with AHLs. Geraniol and eugenol were also found to be the potent inhibitors of *A. baumannii* biofilms, with MICs of 3.24 mM and 6.08 mM, respectively [55]. *In silico* docking indicated an efficient binding of both compounds with the *csuE* protein of archaic pilus ‘Csu’ assembly (binding energy −5.22 kcal/mol and −4.13 kcal/mol, respectively), causing a decrease in expression of the *csuE* gene and antibiofilm action and preventing the assembly of mature pilus. The modelled complex with GER was stabilized by hydrophobic interactions with phenylalanine, valine, and alanine and a hydrogen bond with serine. It was thus postulated that the binding of eugenol and geraniol with the *csuE* subunit may prevent the surface adhesion of *A. baumannii* cells and hinder the formation of mature biofilm structures.

#### 3.2.3. Citronellol

Citronellol (3,7-dimethyloct-6-en-1-ol) (CIT), also known as dihydrogeraniol, of moderate lipophilicity, exhibited bacteriostatic and bactericidal activity against G+ bacteria but a slightly weaker effect against G- species. Nevertheless, CIT still showed a remarkable antimicrobial and antibiofilm action towards *E. coli* [89]. CIT and geraniol (and their oxidated forms) eradicated *E. coli*, *S. aureus*, and *Corynebacterium glutamicum* bacterial strains [90]. The potential of citronellol to inhibit the NorA efflux pump in *S. aureus* 1199 and 1199B was also studied [91]. It was postulated that in the studied system, CIT did not exhibit direct antibacterial activity; however, it enhanced the effect of norfloxacin and enhanced fluorescence emission of EtBr inside the cells of the tested strains. This effect pointed to inhibition of the NorA efflux pump by CIT and an increase in the bacterial membrane permeability. The interactions with NorA were analysed *in silico* to show that CIT binds to the NorA efflux pump receptor in a similar way to chlorpromazine (a drug that inhibits the access of calcium ions to Ca^2+^-dependent ATPases).

Citronellal (3,7-dimethyloct-6-enal) is an aldehyde derivative of CIT that shows high antimicrobial activity due to the presence of nucleophilic carbonyl oxygen (which can participate in the formation of strong hydrogen bonds, e.g., with glycine) [92,93]. Molecular docking also showed hydrophobic interactions and different types of bonding interactions (like hydrogen bonds, π–alkyl, alkyl–alkyl, π-π, π–cation) between bacterial proteins and a part of the citronellal molecule that is also present in CIT (carbon–hydrogen interactions and alkyl interactions with valine, isoleucine, and alanine (binding energy of −5.7 kcal/mol)) [93].

#### 3.2.4. Farnesol

Farnesol (3,7,11-trimethyl-2,6,10-dodecatrien-1-ol) (FAR)—a sesquiterpene alcohol—is a major component of essential oils formed in, e.g., lily of the valley (*Convallaria majalis*), citrus (*Citrus* sp.), or nutmeg (*Myristica fragrans*). FAR is known as a quorum-sensing molecule that affects many stages of yeast *Candida albicans* biofilm development [94]. It was postulated that FAR antifungal activity (*E*,*E*-isomer only [95]) is based on targeting reactive oxygen species production, the induction of cell apoptosis, and the modulation of virulence factors. Other studies on the effect of *E*,*E*-farnesol on G+ and G- bacterial strains showed that only G+ species were sensitive to this treatment [96,97,98]. Moreover, FAR sensitized the cells of *S. aureus* towards gentamycin [97] and modulated the effect of a non-inhibitory β-lactam antibiotic against *S. aureus* MRSA [96]. *In silico* docking suggested the binding affinity of FAR molecules with proteins (IcaA and Sortase (Srt)) responsible for bacterial adhesion in biofilm production (QQ effect of FAR). Ethanolic formulations of FAR inhibited the formation of biofilms and disrupted mature biofilms of *S. aureus* and *P. aeruginosa*, including their G+/G- polymicrobial biofilms, by directly killing the microorganisms and facilitating biofilm detachment [99]. It was also shown that FAR can destroy the structure of *S. epidermidis* biofilm [100]. However, the tolerance of *S. aureus* to antimicrobials was significantly enhanced in the presence of exogenously supplemented FAR or FAR secreted by *C. albicans* in a mixed biofilm of *S. aureus* and *C. albicans* [101]. The effect was attributed to upregulation of drug efflux pumps and farnesol-induced oxidative stress.

#### 3.2.5. Phytol

Phytol [(5*R*,9*R*)-5,6,7,8,9,10,11,12-octahydro-1,6-secoretinol] (PHY) is an acyclic hydrogenated diterpene alcohol abundantly found in nature. It is produced by almost all photosynthetic organisms (a component of chlorophyll). PHY is mainly used as a fragrance and an intermediate in the synthesis of vitamins (E and K_1_). It was postulated that as another linear terpene, PHY has the lipophilic capability to cross cell membranes; can be immune-modulating and cytotoxic (autophagy- and apoptosis-inducing); and has antioxidant, anti-inflammatory, and antimicrobial properties [102,103]. Phytol was found in plant extracts that showed inhibitory activity against isolated bacterial strains and algae [104,105,106,107,108]. Treatment with PHY increased the level of intracellular reactive oxygen species in *P. aeruginosa* and lowered the level of reduced glutathione [109]. It was also postulated that phytol has similar binding affinities for the proteins 6GJ1 (T6SS, *E. coli*) and 5KDR (carboxyltransferase, *S. aureus*) (−5.06 kcal/mol and −5.51 kcal/mol, respectively) through π-σ, π–alkyl, and alkyl interactions (with 6GJ1); van der Waals and π-σ interactions; and alkyl and π–alkyl interactions (with 5KDR) [110]. Interestingly, despite the antagonistic interactions between phytol and norfloxacin (molecular competition for the same binding sites in carboxyltransferase) in *S. aureus* [110], the same combination displayed a clinically relevant enhancement of the antibiotic action against *Salmonella* spp. [111] and *E. coli* [110]. The effect was attributed to an increase in membrane permeability caused by PHY. It was also reported that isophytol (3,7,11,15-tetramethylhexadec-1-en-3-ol) inhibited the growth of *S. enterica*, *S. aureus*, and *Aspergillus niger*, and when used as an adjuvant, it enhanced the action of *Ginkgo biloba* polyprenols against *S. enterica* [112].

Phytol also has strong antibiofilm properties. Comparative studies on *K. pneumoniae* biofilms showed that MIC values of PHY were similar to those of glycitein and α-terpinene of (0.125 mg/mL) and caused a ~66% reduction in EPS (similar antivirulence activity as camphene) [113]. PHY showed the highest anti-adhesion activity against carbapenem-resistant β-lactamase-positive *K. pneumoniae* (~55%) and caused a significant altteration of the biofilm architecture. It also inhibited quorum sensing and biogenic amine production in G- *P. fluorescens* PF12 [114]. A reduction in key genes related to histamine synthesis (hisD and hisC) was observed. Moreover, *in silico* modelling revealed that PHY can interact with key residues in the LuxI-type protein and can block the synthesis of AHLs. In combination with cefotaxime against *A. baumannii*, PHY effectively inhibited biofilm formation [115]. The anti-quorum-sensing activity of phytol (inhibition of biofilm formation, pyocyanin production, and motility) was documented against the *P. aeruginosa* PAO1 [116] and *C. violaceum* (ATCC 12472) wild-type strain [117]. The anti-QS action of PHY was also proven in tests with *Serratia marcescens* (decreasing levels of biofilm formation; lipase and hemolysin production by the downregulation of fimA, fimC, flhC, flhD, bsmB, pigP, and shlA gene expression; inhibition of swarming motility; and EPS production) [118]. The promising quorum-quenching activity of a PHY-containing *Plectranthus barbatus* EOs was also recorded for *L. monocytogenes* (ATCC 13932), *S. enterica* subsp. enterica serovar *Typhimurium* (ATCC 25241), and *E. coli* (ATCC 11775) [117].

## 4. Polymeric Materials with Antibacterial Properties Resulting from Their Modification with Essential Oils

Different aspects of antimicrobial activity of various components of essential oils have been collected in Table 1. Antibacterial systems based on polymers containing such essential oils can take the form of nanovessels, nanofibers, active films, hydrogels, and polymer scaffolds [119,120,121,122]. The specific structure of these systems can determine their properties and the range of potential applications. These topics are discussed in the following subsections.

### 4.1. Nanoencapsulation

**Figure 8:**
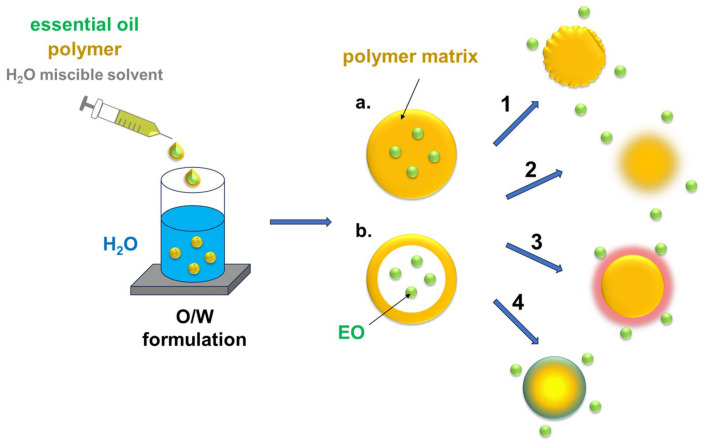
Preparation of polymeric nanoparticles (a: nanospheres and b: nanocapsules) through oil-in-water (O/W) formulations and potential release mechanisms of the EOs’ content under different stimuli (1: mechanical, 2: chemical, 3: thermal, 4: solvent diffusion).

Although the formation of water-dispersible nanoemulsions (10–100 nm) can be a good method to increase the bactericidal action of essential oils (or their isolated components) against microbial pathogens [123,124,125], their micro-/nanoencapsulation in polymeric systems is more efficient in overcoming the challenges related to their poor solubility in water (or dispersion of the nonpolar molecules in an aqueous environment), volatility, low bioavailability, susceptibility to oxidation, thermolability, and instability with variations in pH and exposure to light [126,127,128]. Encapsulation of the bioactive compounds can also improve their bio-distribution and enable controlled release [129]. Nanotechnology provides platforms (nanocarriers, nanoemulsions, and liposomes) that can be used for effective administration of EOs in medicine and materials engineering. This approach facilitates prolonged release to promote antimicrobial action over time and prevents interactions that could impair this effect [130,131,132,133]. Nanoencapsulation strategies for loading EOs in natural or synthetic polymeric matrices through oil-in-water (O/W) formulations can lead to the formation of two types of nanoparticles: nanocapsules with an active material confined in a polymer shell and nanospheres with an active material dispersed in a polymer matrix (Figure 8). Synthetic polymer components can be prepolymerized prior to the encapsulation step and employed in nanoprecipitation/emulsification/coacervation or polymerized during the process (in situ polymerization). The release of volatile phytocompounds from polymer nanoparticles can occur via slow diffusion or under the influence of chemical, mechanical, and thermal stimuli.

Loading the active components in nanosized drug delivery systems with the purpose of enhancing their bioactive properties is widely exploited for pharmaceutic and cosmetic applications (various topical formulations, including topical gels and lotions) [134,135]. It has been found that various types of liposomal preparations are effective against both G+ and G- species [136]. The key to maintaining the antibacterial efficacy of the free form after nanoencapsulation is the size and morphology of the nanocontainers [137,138,139]. Optimization of these parameters can enhance the potency of EOs and improve the bioavailability, biocompatibility, and shelf life without losing their beneficial properties (especially the ability to penetrate and cross the bacterial cell membrane). Comparative studies on antimicrobial activity of eugenol and linalool encapsulated in polylactic acid (PLA) against *E. coli*, *S. enterica*, *S. aureus*, and *L. monocytogenes* showed that the capsules continued to release the active species for up to 40 days [140]. Inhibition zones of a relevant size were formed with the encapsulated EUG in the tests against *E. coli* (60 mm) and LIN against *Salmonella* (32 mm).

Slow release of EOs from nano/microcapsules may also improve the bacteria-repellent performance of polymer coatings. For example, EUG in poly(lauryl acrylate) nanocapsules entrapped in an interpenetrating *co*-poly(methyl methacrylate/dimethylacrylamide) network significantly reduced the amount of *K. pneumoniae* and methicillin-resistant *S. aureus* (MRSA) on the samples’ surface [141]. Eugenol can be also transported in the corona of hybrid particles, e.g., silica nanoparticles surrounded with triblock Pluronic F-127 arms [142]. The emulsions of the hybrid nanocarriers were stable in H_2_O when the ratio between the weight fractions of Pluronic F-127 and EUG was > 1.5.

The use of uniform linalool-based lipid nanocapsules (LNCs) loaded with resveratrol (LIN-LNC-RES) (size of ~35 nm), prepared by the phase inversion temperature method, being stable at 4 °C for 3 months, was proposed as a synergistic strategy against *S. aureus* MRSA [143]. The LNC consisted of an oily core surrounded by a lipophilic surfactant (lecithin) and a pegylated non-ionic surfactant (a mixture of free polyethylene glycol 660 and polyethylene glycol 660 hydroxystearate) with a rigid outer shell. LIN-LNC-RES provided a controlled release of RES over 24 h and was effective against biofilms. Geraniol-loaded Pluronic^®^ F-127 nanoparticles (size distribution of 26–412 nm, depending on the ratio of GER to Pluronic^®^ F-127), prepared by flash nanoprecipitation, sustainably released GER over 24 h and inhibited the growth of *S. enterica Typhimurium* and *E. coli* O157:H7 on spinach leaves at a concentration of 0.25 and 0.2 wt.%, respectively [144]. GER encapsulated in spherical polycaprolactone (PCL) capsules prepared by miniemulsion polymerization (encapsulation efficiency of > 95%, average capsule size of 148 nm, and polydispersity of 0.12) remained stable for 60 days in aqueous suspensions at 4 °C [145]. Thermogravimetric analysis showed the release of encapsulated GER at temperatures of approximately 100 °C higher than the volatilization temperature of the pure compound. Encapsulation of lemongrass essential oil containing GER in aggregated spherical chitosan nanoparticles (average hydrodynamic size of 175–235 nm, depending on the EOs-to-chitosan ratio) also increased its release temperature by about 120 °C [146]. The release kinetics were dependent on time and pH, characterized by a rapid initial release of EOs and, in later stages, a constant rate of its migration from the chitosan shell.

**Figure 9:**
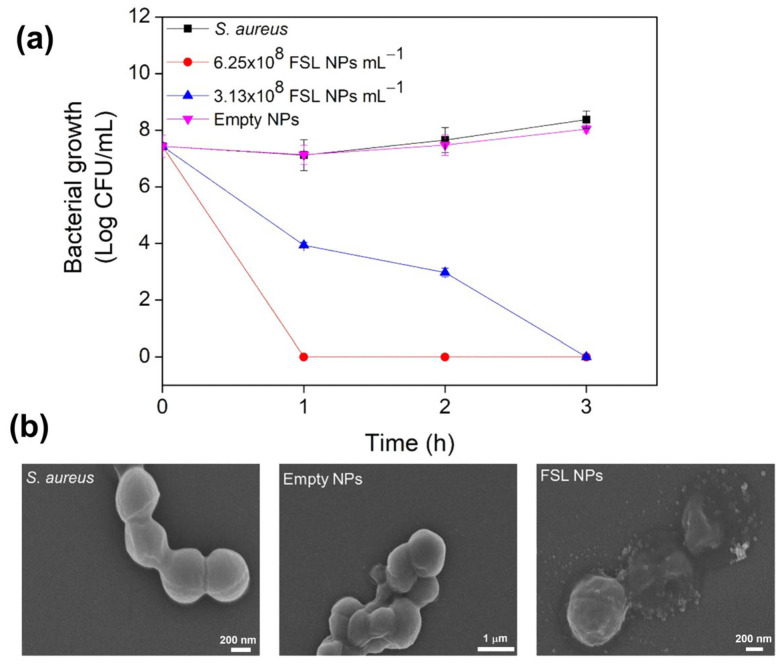
(**a**) Time-killing kinetics illustrating the effect of farnesol-loaded nanoparticles; (**b**) SEM images of *S. aureus* cells (without any treatment, incubated with empty NPs, and with farnesol-containing NPs (FSL NPs) [149].

Amphiphilic molecules of farnesol are long enough to form large supramolecular systems, but, as other components of essential oils, FAR can be protected inside polymeric nanovessels to enhance its contact with microbial biofilms and planktonic cells under controlled release conditions [7,147,148]. FAR encapsulated in a biocompatible monobutylester of poly(methylvinylether/maleic) acid using high-intensity ultrasound completely eliminated planktonic *S. aureus* in less than 3 h (Figure 9), without inducing resistance [149]. Even at low concentrations, FAR nanocapsules inhibited the formation of *S. aureus* biofilm and eradicated the mature biofilm.

[Poly(dimethylaminoethyl methacrylate)-*b*-poly(dimethylaminoethyl methacrylate)-*co*-butyl methacrylate-*co*-propylacrylic acid] micelles loaded with FAR (~22 wt.%) were successfully used against biofilms of *S. mutants* [150,151]. FAR release was pH-dependent (t_1/2_ = 7 h at pH 4.5 and 15 h at pH 7.2). Interactions with biofilms involved the disruption of the insoluble glucan envelope and enhanced the insertion of the active species into the cell membrane. Treatment with drug-loaded nanoparticles weakened the mechanical stability of the biofilm. FAR loaded into biodegradable polymeric poly(DL-lactide-co-glycolide) nanoparticles by means of the emulsion evaporation method decreased the growth of *C. albicans* more effectively than neat FAR [152].

**Figure 10:**
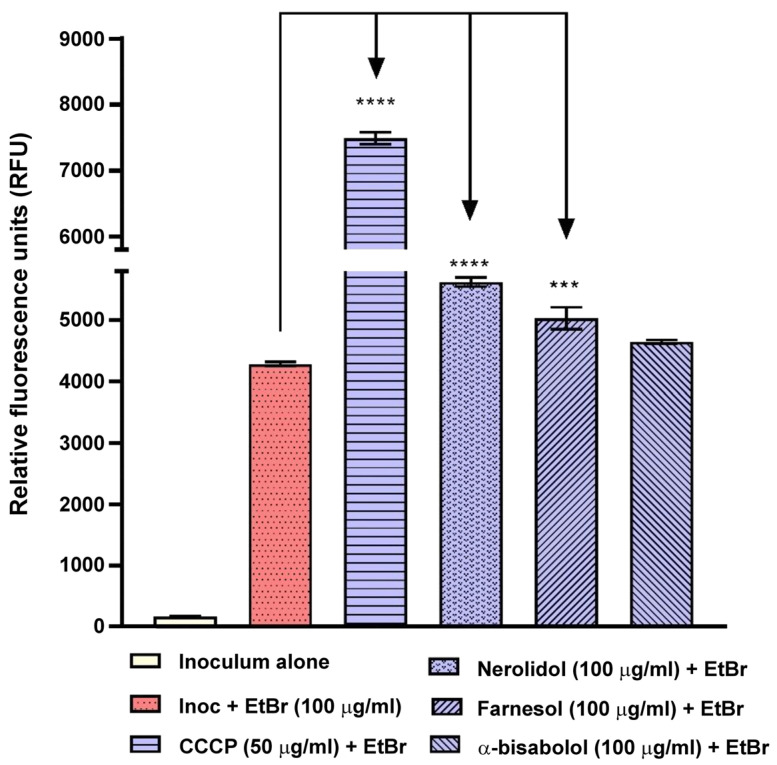
Evaluation of MepA efflux pump inhibition in *S. aureus* K2068, treated with EOs (nerolidol, farnesol, and α-bisabolol) at 100 µg/mL, compared to the effect of ethidium bromide (EtBr); inoculum (Inoc); and a standard efflux pump inhibitor, carbonyl cyanide 3-chlorophenylhydrazone (CCCP), **** = *p* < 0.0001 vs. Inoc + EtBr; *** = *p* < 0.001 vs. Inoc + EtBr [153].

As was previously mentioned, interactions with the system of efflux pumps in the microbial cell membrane are crucial for the effective eradication of planktonic bacteria and biofilms. Encapsulation limits effective contact with bacteria, and interactions with EPs are based only on the action of molecules released from nanovessels. Nevertheless, it has been shown that sesquiterpenes (nerolidol, farnesol, and α-bisabolol, used alone or in liposomal nanoformulations made up of 1,2-dipalmitoyl-sn-glycero-3-phosphocholine, cholesterol, and distearoylphosphatidylcholine) can affect the NorA, Tet(K), MsrA, and MepA efflux systems in multidrug-resistant *S. aureus* strains [153]. The liposome/farnesol system was the most promising as it acted on both NorA present in *S. aureus* 1199B and MsrA in the *S. aureus* RN4220 strain (Figure 10).

As mentioned earlier, EOs interactions with efflux pumps help other antibiotic substances enter the cells. Thus, studies on the encapsulation of phytochemicals and drugs in liposomal nanoparticles have been undertaken. Farnesol combined with ciprofloxacin was used in liposomal form (entrapped in nanovessels formed with 1,2-dipalmitoyl-*sn*-glycero-3-phosphocholine and cholesterol) to eradicate *P. aeruginosa* biofilms [154]. In the presence of FAR, the concentration of ciprofloxacin required to inhibit biofilm metabolism could be reduced. Interestingly, the release of ciprofloxacin from liposomes was more effective when it was encapsulated with farnesol, whereas FAR release was lower in the hybrid system than in liposomes containing farnesol alone. The effectiveness of geraniol and farnesol in combination with colistin (a last-resort antibiotic) and encapsulated in water-dispersible lipid nanoparticles in combating *E. coli* J53 MCR-1 to prevent the development of resistance was also investigated [155]. Both GER and FAR restored the sensitivity of *E. coli* J53 MCR-1 to colistin with the same efficacy; although, with an EC_50_ of 2.69 mg/L, farnesol was more potent than geraniol (EC_50_ = 39.49 mg/L). Farnesol-loaded nanoparticles at 60 mg/L enhanced the effect of colistin in increasing inner-membrane permeability and also increased its efficacy.

Farnesol and essential oils extracted from eucalyptus leaves (*E. globulus* L.) and lemon peel (*C. limon* L.) were also enclosed in silica nanocapsules obtained by the sol–gel method (water-in-oil-in-water—O/W/O—multiple emulsions using retinol and oleic acid) [156,157]. The systems were tested for controlled release of the bioactive compounds in the gas phase and in ethanol solutions. The capsules proved effective in ensuring prolonged, controlled release of phytochemicals, and the corresponding release profiles depended both on the parameters used during the capsules’ preparation and on the chemical properties of the terpenoids.

### 4.2. Nanofibers

**Figure 11:**
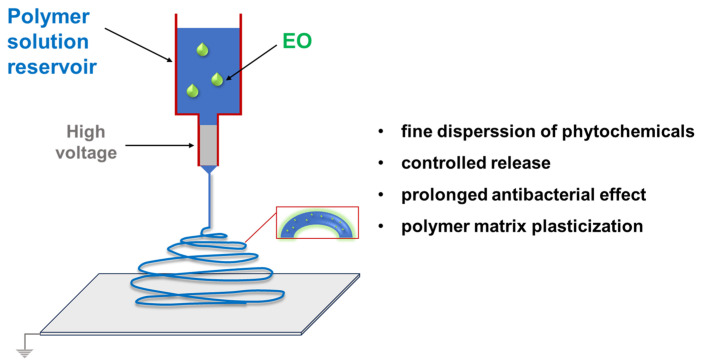
Formation of electrospun nanofibers as a method for encapsulation of EOs and its controlled release.

Electrospinning has become one of the most promising techniques for producing nanofibers for various applications, including environmental remediation and biomedicine. Biopolymers are an ideal material for the preparation of biocompatible and biodegradable electrospun nanofibers. However, they often require modification by blending with various additives (nanoparticles, cross-linking agents, bioactive molecules, etc.) to improve their mechanical and antibacterial properties. Essential oils can be used as bioactive components and modifiers in electrospun nanofibers [158]. On the other hand, encapsulation of EOs in nanofibers facilitates controlled release of the phytocompounds (Figure 11).

For example, linalool in electrospun and solution-blow-spun (SBS) poly(lactic acid) nanofibrous membranes (at concentrations of 10, 15, and 20 wt.%; fiber diameters of 176–240 nm) acted as a classic PLA plasticizer, lowering the temperatures of characteristic phase transitions [159]. The time required for the release of 50% LIN (t_1/2_) at 35° C depended on the technique of nanofiber preparation and decreased with increasing the content of LIN in PLA. Thinner electrospun nanofibers released LIN much faster (76–575 s) than SBS nanofibers (291–1645 s). Loading limonene and linalool in electrospun poly(vinyl alcohol) (PVA) nanofibers did not cause a significant change in the diameter of fibers (~158 nm and ~175 nm for pure PVA and the matrix with encapsulated essential oils, respectively) [160]. The hybrid samples effectively eradicated *C. albicans* and *Candida tropicalis* biofilms (>90%) while causing negligible cytotoxic effects. Encapsulation of lavender essential oil (main components: linalool (~35%) and linanlyl acetate (~26%)) in electrospun gelatin nanofibers (0, 2.5, 0.5, and 0.10% *v*/*v*) resulted in an increase in nanofiber thickness (431 to 705 nm) and the content of the phytochemicals (4.7–14.6%) with increasing the concentration of EOs in the matrix [161]. The nanofibers were uniform and continuous, but the plasticizing effect caused by the presence of EOs affected their mechanical properties (a significant decrease in Young’s modulus and stiffness). The antibacterial activity of the nanofibers against *S. aureus*, *Bacillus cereus*, *E. coli*, and *Salmonella typhimurium* was also investigated.

Hydrophobic electrospun polyvinylidene difluoride (PVDF) nanofibers (wetting angle of > 130°) loaded with essential oils (thymol, eugenol, linalool, cinnamaldehyde, and carvacrol) had diameters ranging from 570 to 900 nm [162]. Samples containing thymol and EUG displayed antifouling activity against *E. coli* ATCC 25922 and *S. aureus* ATCC 25923. The hydrophobicity and antimicrobial activity make the materials suitable for preparation of filtration membranes. Essential oils have also been applied in complex technologies, such as the incorporation of various active ingredients in multi-channel electrospun fibers to enhance their antibacterial performance [163]. A side-by-side electrospinning of silver nanoparticles/PCL and lavender oil (main component linalool)/cellulose acetate (CA) mixtures was performed using a specially designed Janus spinneret. This resulted in a hybrid PCL/CA Janus nanobelt with Ag NPs on the PCL side and lavender oil on the CA side. Antibacterial tests showed inhibition zones with a diameter of approximately 14 mm due to the synergistic antibacterial action of Ag NPs and linalool against *E. coli.*

Interesting results were obtained with essential oil components introduced into the electrospinning solution as inclusion complexes with cyclodextrin hosts. Cyclodextrins are natural oligosaccharides with toroidal structures consisting of six (α-CD), seven (β-CD), or eight (γ-CD) D-(+)-glucose subunits linked by α-1,4 glycosidic bonds. Due to their intrinsic capability to accommodate small hydrophobic molecules inside the hydrophobic central cavity lined with skeletal oxygen atoms and methine units, they are valuable carriers for water-insoluble drugs [164]. β-cyclodextrin (β-CD) inclusion complexes with geraniol (β-CD/GER) were electrospun as polymer-free free-standing nanofibrous webs that were effective against *E. coli* and *S. aureus* [165]. It was observed that the uniform and bead-free β-CD/GER nanofibers had higher antioxidant activity compared to pure GER. Polymer-free fibers attained much higher geraniol loading (~11%, *w*/*w*; ~60–90% of geraniol was preserved in the nanofibers) when compared to electrospun PVA nanofibers containing CD/geraniol-IC (~5%, *w*/*w*). The release profile depended on the structure of the cyclodextrin employed in the process (three types of β-CDs modified with hydroxypropyl and methyl groups (HPβCD, MβCD, and HPγCD) were used). Release tests [short-term (3 h) temperature dependent (37 °C, 50 °C, and 75 °C) and long-term in the open air (50 days, at RT)] revealed that the most effective were nanofibers prepared with the most hydrophobic complex with MβCD. They released less GER compared to HPβCD/geraniol-IC-NF and HPγCD/geraniol-IC-NF. The same host system, but with linalool as the guest molecule, showed similar antibacterial properties against *E. coli* and *S. aureus* [166]. The maximum loading of LIN in β-CD was 12% (*w*/*w*) (45–89% of LIN retained in the nanofibers). Remarkably, the nanofibers containing both geraniol and linalool had very fast dissolving characteristics in water. It has also been shown that inclusion complexes composed of β-cyclodextrin and terpenes can be successfully employed with gentamicin, exhibiting a synergistic action against *E. coli* [167]. The effect was drug-specific, and the same complex in combination with norfloxacin displayed antagonistic or neutral effects against the tested strains.

CD-based inclusion complexes obtained with LIN and GER retained their antimicrobial activity when used as additives in electrospun polymeric nanofibers. For example, the inclusion complex of geraniol and β-cyclodextrin loaded in an acacia gum/PVA polymer matrix using electrospinning had little effect on fiber diameter (121 nm and 142 nm for the GER-loaded and neat nanofibers, respectively) [168]. Prolonged release of geraniol from the nanofibers was observed for up to 150 min, and it efficiently inhibited 80% of *C. albicans* and *Nakaseomyces glabrata* fungal growth. The GER-loaded nanofibers were also effective against the mature biofilm (85% eradication at 10 mg/mL of nanofiber) and showed no cytotoxicity in tests with Chinese hamster ovary cells, making the material suitable for use against sessile fungal infections. The β-CD/EUG inclusion complex was incorporated into PLA electrospun fibers, used in combination with a super absorbent polymer (SAP) and filter paper to form a water-absorbent antibacterial mat [169]. A stable EUG release profile was observed for up to 40 min, inhibiting the growth of *S. aureus*, *E. coli*, *P. aeruginosa*, *L. monocytogenes*, *Bacillus pyocyaneus*, and *S. typhimurium*.

Electrospun gelatin mats made of defect-free cylindrical fibers (diameters of ~315–480 nm), containing inclusion complexes of β-CD and cinnamaldehyde, limonene, and eugenol, slowly released EOs over 60 days at ambient temperature [170]. The mats exhibited similar antifungal activity against *Aspergillus niger* at 5% concentration of the EOs, but their antibacterial properties against *S. aureus* and *E. coli* depended on the type of the guest phytocompound. Mats containing EUG showed up to 96.5% of the radical scavenging activity, while those with cinnamaldehyde featured an exceptional antibacterial activity (an inhibition zone of ~28 mm). The relatively low activity of β-CD/limonene mats can be explained by the tight binding of limonene molecules in the cavity of the β-CD host. Although the presence of inclusion complexes reduced the tensile strength and elongation at break of the gelatin mats, their melting point was slightly improved. This system has the potential to be used in active antibacterial food packaging (see Section 4.4).

Linalool and geraniol hosted inside gamma–cyclodextrin (γ-CD) molecules were enclosed in pullulan electrospun nanofibers [171]. Approximately 74 to 77% of the terpenoids remained inside the inclusion complexes (the initial EOs content was ~11% (*w*/*w*)). The presence of the cyclodextrin host was crucial for achieving this effect, as only about 15–23% of the uncomplexed EOs were retained. The hybrid nanofibers increased the long-term storage stability of EOs (after 1 month, approximately 48–64% of terpenoids were still present in the complexes, while only about 0.3 to 4.5% remained in pullulan/EOs nanofibers). Thermal volatilization of essential oils increased from about 119–139 to 239–292 °C after the complexation and encapsulation in the nanofibers. Samples containing EOs-γCD displayed an effective antibacterial activity against *S. aureus* and *E. coli* (the growth of bacterial strains was prevented for up to 10 h).

### 4.3. Nanocomposites and Blends

Various types of polymer matrices can be used as carriers of essential oils, both in free form as components of polymer blends and with the bioactive molecules chemically bound to macromolecular structures. It was shown that the essential oils (both biophenols and linear terpene alcohols) can act as natural additives/modifiers of polymer materials. It should be noted that the antimicrobial performance of EOs physically blended with polymers is strongly influenced by both their structure and phase-separation phenomena.

#### 4.3.1. Physical Blends

**Figure 12:**
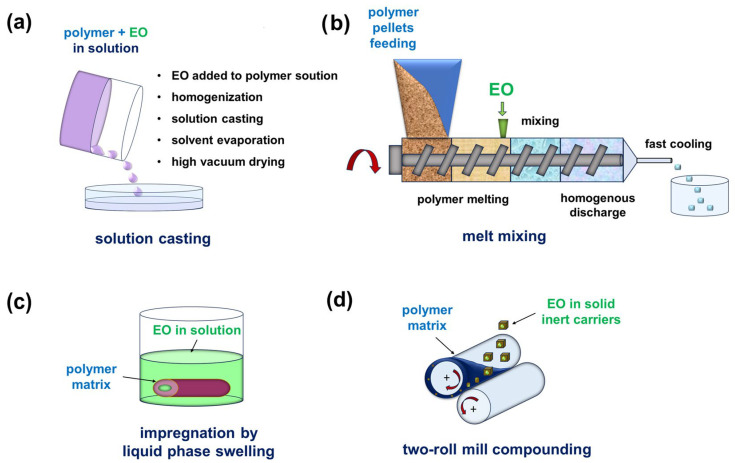
Exemplary methods for the formation of EOs/polymer physical blends: (**a**) solution casting; (**b**) melt mixing; (**c**) impregnation; and (**d**) two-roll mill compounding.

Various methods can be applied for physically blending essential oils with polymers (Figure 12). For example, the range of composites with incorporated eugenol includes polyesters, polyurethanes, polyacrylates, epoxies, and polyolefins [172]. Of particular importance is the use of EUG to modify natural biopolymers or “green” synthetic polymers, such as a mixture of chitosan and poly(vinyl alcohol), which effectively prevented infections associated with *S. aureus* and *P. aeruginosa* biofilms [173].

Hydroxyethylcellulose and hydroxypropylcellulose, combined with polyethylene glycol 400, have been used as mucoadhesive films for dental applications and for mucosal drug delivery [174]. The incorporation of clove extract (main component: EUG) and lavender and grapefruit essential oils resulted in significant antimicrobial activity and a prolonged therapeutic effect against a wide spectrum of microorganisms (*S. aureus* ATCC 25923, *S. epidermidis* ATCC 13076, *Streptococcus mutans* ATCC 25175, *Streptococcus mitis* ATCC 6249, *E. faecalis* ATCC 29212, *E. coli* ATCC 25922, *K. pneumoniae* ATCC 13883, *P. aeruginosa* ATCC 27853, *Proteus mirabilis* ATCC 29906, *B. cereus* ATCC 11778, and *C. albicans* ATCC 10231). It was found that approximately 90% of EUG was released in the final phase of film dissolution (360–420 min).

Antibacterial textiles modified with essential oils can be obtained using various techniques, but their long-term antimicrobial stability and mechanical properties may vary. For example, herbal extracts containing eugenol, germacrene, and phytol were applied to the cotton fabric directly or through micro-encapsulation, resin cross-linking, and combinations thereof [175]. The treated samples showed good antibacterial activity against *S. aureus* and *K. pneumoniae*, regardless of the application method. The washing durability was good (up to 15 washes), except for the fabric obtained by direct application. The directly treated and the micro-encapsulated fabrics showed a slight decrease in tensile strength and crease recovery angle, respectively. Another modification method is functionalization through diffusion. Fabrics made of polyethylene terephthalate (PET) were functionalized with thymol, geraniol, cinnamaldehyde, ortho-vanillin, and para-vanillin at 130 °C and under high pressure [176]. The thermal stability of the resulting materials (except for the blend with thymol) exceeded the diffusion temperature of the volatile substances, and the products exhibited antibacterial activity against *S. aureus* and *K. pneumoniae*. Samples containing O-vanillin and cinnamaldehyde also featured good wash durability. However, this process cannot be applied universally. Both the polymer resin and the volatile components should have similar Hildebrand solubility parameters (δ).

Studies on the effect of citronellol, eugenol, and linalool contained in poly(ethylene vinyl acetate) copolymer (EVA) films revealed that the addition of phytocompounds caused plasticization of EVA and affected its mechanical properties (15% decrease in elastic modulus, 30% decrease in tensile strength, and 10% increase in elongation at break) [177]. An evaluation of the antimicrobial and antibiofilm activity of the hybrid blend against *L. monocytogenes*, *S. aureus*, *S. epidermidis*, *E. coli*, and *P. aeruginosa* (single- and dual-species tests) showed that EVA/EUG and EVA/CIT (7% wt. EOs content) most efficiently inhibited bacterial growth (by 15–30% and 30–60%, respectively, after 24–48 h of incubation; the inhibition effect decreased after 240 h). However, in dual-species tests conducted with *S. aureus* and *E. coli*, better results were obtained with EVA/EUG than with EVA/CIT. An assessment of changes in the physical properties of the polymer matrix (thermostability, temperatures of glass transition, crystallization, and melting (and their enthalpy), changes in the polymer morphology and mechanical properties (tensile strength and elongation at break), film opacity), and its antibacterial activity against *E. coli* and *S. aureus* was also made for polylactide films plasticized with geraniol and cinnamaldehyde [178]. Both compounds provided the desired antimicrobial effect, with cinnamaldehyde being more effective than GER. Geraniol was also incorporated by extrusion processing into an agar-based hydrogel, which was evaluated for its activity against *S. epidermidis*, *P. aeruginosa*, *S. aureus*, and *E. coli* [179]. The resulting biocompatible materials had high swelling capacity and shape fidelity, which makes them suitable for printing bioinks. It should be noted that the modification with geraniol also caused a significant decrease in the viability of L929 mouse fibroblast cells (cytotoxic effect).

**Figure 13:**
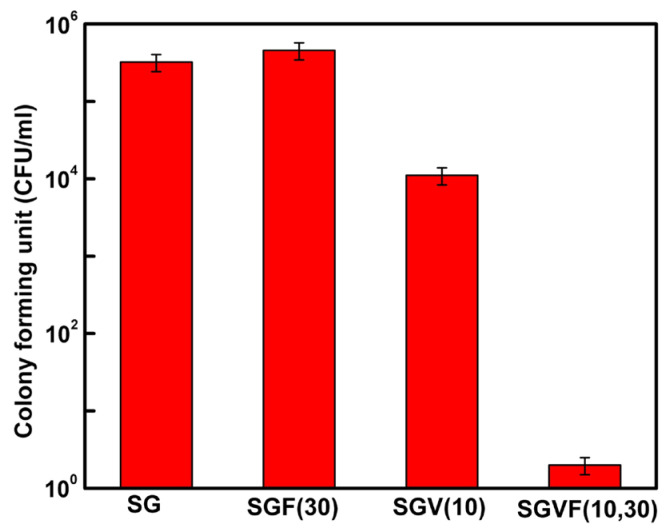
The antibacterial effect obtained by coating Ti rods with thin sol–gel films containing farnesol (SGF) and vancomycin (SGV) alone and their combination (SGVF) against MSSA [182]. Reprinted from Biomaterials, 215/1, Bhattacharyya, S.; Agrawal, A.; Knabe, C.; Ducheyne, P., Sol gel silica controlled release thin films for the inhibition of methicillin-resistant *Staphylococcus aureus*, 509–517, Copyright (2014), with permission from Elsevier.

There are few reports on macromolecular systems containing farnesol and phytol as antibacterial agents. FAR is often used as an antifungal agent against *C. albicans*, and incorporating FAR into a PMMA matrix may be an effective strategy for reducing fungal biofilm formation on dental materials [180,181]. It has been also demonstrated that FAR can reduce the growth rate of bacterial biofilms on polymer surfaces and enhance the efficacy of other drug therapies. For instance, complete inhibition of both methicillin-sensitive and methicillin-resistant *S. aureus* biofilms was achieved using silica sol–gel coatings comprising 30 wt.% of FAR and 10 wt.% of vancomycin (Figure 13) [182]. Phytol was present in *Spondias pinnata* extract, which was conjugated with chitosan for use in anticancer and antibacterial applications [183]. Antibacterial activity of the hybrid against *E. coli* (MTCC 452), *Salmonella typhi* (MTCC 733), *K. pneumonia* (MTCC 39), and *P. aeruginosa* (MTCC 1688) was noted, with inhibition zones measuring approximately 16, 19, 17, and 19 mm, respectively.

Another method of introducing EOs into polymer matrices is impregnation through liquid phase swelling. For example, linalool and 2-hydroxy-4-methoxybenzaldehyde (HMB) were introduced into silicone Foley catheters (SFCs) via solvent swelling to prevent urinary tract infections caused by *Proteus mirabilis* [184]. The impregnation efficacy and stability were good and did not change the catheter surface topography. An in vitro quantitative biofilm biomass assay demonstrated 87% and 84% inhibition of *P. mirabilis* crystalline biofilm (a type of biofilm containing embedded Mg^2+^ and Ca^2+^ phosphate crystals) on the surface of SFCs impregnated with LIN (350 μg/cm^3^) and HMB (120 μg/cm^3^), respectively. The sustained release of the phytocompounds over 30 days provided long-term inhibitory action towards crystalline biofilms in the impregnated samples.

Essential oils can be also introduced into polymeric matrices using solid inert carriers. Linalool, 4-allylanisole, and trans-anethole were physically immobilized in wood flour (WF), talc, and molecular sieves and subsequently incorporated as additives into a low-density polyethylene (LDPE) matrix using a two-roll mill compounding technique [185]. The highest antimicrobial effect was observed in systems containing 4-allylanisole and WF. Under those conditions, *E. coli* was more inhibited than *S. aureus*. The least compatible of the tested systems was that consisting of a hydrophilic WF and nonpolar LDPE matrix (poor WF dispersion and low adhesion of the components, clearly visible in SEM micrographs as two separate phases).

#### 4.3.2. Covalent Grafting

**Figure 14:**
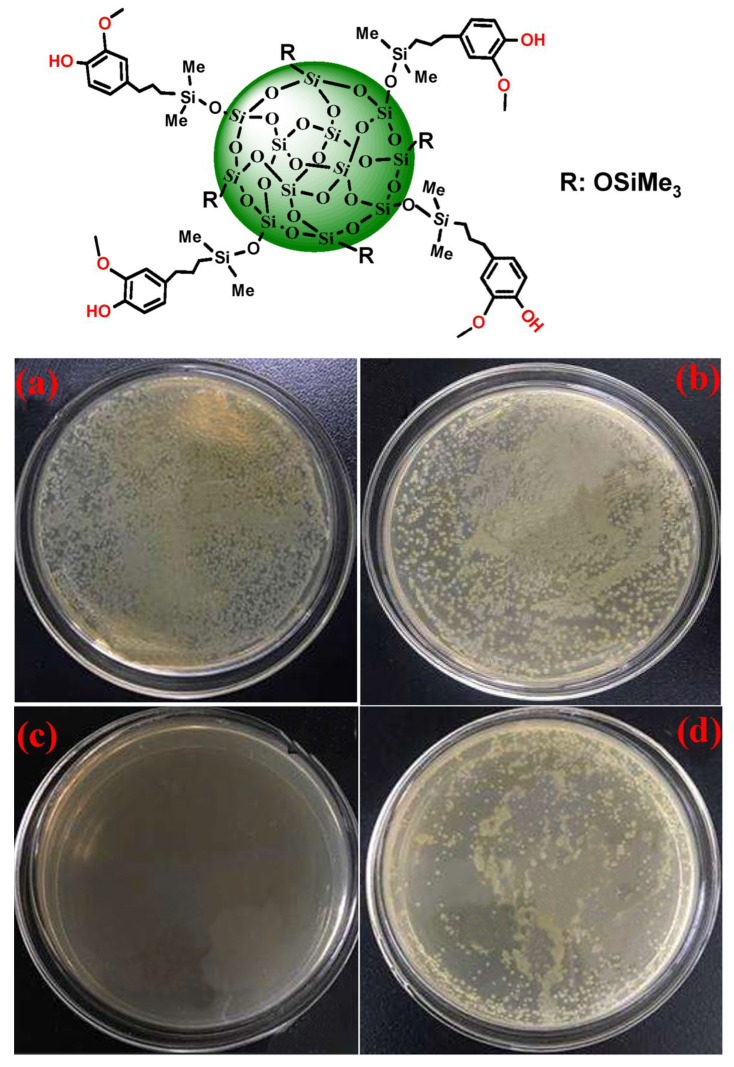
Schematic structure of BPMQ hybrid (top) and optical images of agar plates showing the bactericidal activity of HMQ (**b**), eugenol (**c**), and BPMQ (**d**) in comparison to a blank control (**a**). Adapted from [191].

Antimicrobial phytochemicals also have significant potential as bio-based monomers or precursors in green polymer technologies. EUG-based polymeric materials exhibit enhanced physicochemical characteristics and can be used to generate coatings that prevent bacterial biofilm growth [186,187]. Some of these coatings can also self-heal [188,189], which improves their mechanical stability and makes them less susceptible to microbial settlement. Although covalent bonding is a very effective method of immobilizing phytochemicals in a polymer matrix, it is crucial to employ a modification method that preserves the phytocompound’s most microbiologically active feature. For instance, the grafting of eugenol and linalool to hybrid polysilsesquioxanes via hydrosilylation with trialkoxysilane, followed by a sol–gel reaction and coating of the oligomers onto an activated glass surface, yielded coatings resistant against the settlement of *A. hydrophila* [190] (the adhesion of bacterial cells after 6 days of incubation was 8–12 times lower than in the control group). Consequently, the immobilization of EUG through hydrosilylation of its alkenyl group with hydrogen-containing MQ silicone resin (HMQ; M: monofunctional and Q: tetrafunctional siloxane units) led to the formation of a bio-phenol MQ silicone hybrid (BPMQ) with high thermal stability (T_dmax_ > 420 °C) and lower susceptibility to *E. coli* microbial colony growth (Figure 14) [191].

Transforming EUG into eugenyl methacrylate (EUMA) creates a reactive compound that can be copolymerized with various acrylate or vinyl monomers. For example, EUMA, used as a co-monomer in free-radical emulsion polymerization with dopamine methacrylamide (DMA), yielded poly(DMA-co-EUMA) particles of uniform size and high antibacterial activity against *E. coli* (>90%) [192]. Copolymers of EUMA with 2-hydroxyethyl methacrylate (HEMA) (pHEMA-EUMA) acted as radical scavengers and exhibited increased antimicrobial activity against *S. epidermidis* when the EUMA content was 30% (~20% bacterial growth) [193]. Conversely, using the phenolic function for covalent grafting significantly reduced the antimicrobial potential of EUG. For example, the antimicrobial activity of a polyester derived from the copolymerization of L-lactic acid O-carboxyanhydride and eugenol-containing L-malic acid O-carboxyanhydride was relatively low [194]. The hybrid polymeric material was active against bacteria such as *S. aureus* and *B. cereus*, and to a lesser extent, against *P. aeruginosa*, but no inhibition was observed against *E. coli*. It was also observed that the antibacterial activity of hybrid polymacrolactones against *E. coli* could be modulated by grafting 4-(3-mercaptopropyl)-2-methoxyphenol (a mercapto-derivative of eugenol) in the side chains [195].

Linear terpene alcohols are much less susceptible to the blocking of their hydroxyl groups through chemical reactions. For example, grafting geraniol onto chitosan (a linear polysaccharide obtained by the partial deacetylation of chitin, which is biocompatible and biodegradable and exhibits antimicrobial and antioxidant properties [196]) was proposed as an effective strategy for enhancing its antibacterial properties and simultaneously improving the solubility of GER in water [197]. The antimicrobial activity of chitosan, GER, and chitosan–GER hybrids against *S. aureus* was more prominent than against *E. coli*. In the case of linear terpenoids, it appears that the key factor is maintaining the hydrophobicity of the hydrocarbon chain after modification. The chemo-enzymatic oxidation of unsaturated bonds in the alkenyl chains of terpenoid alcohols (CIT and GER) did not change their overall antimicrobial effect (at MIC and 2MIC) against *E. coli*, *S. aureus*, and *Corynebacterium glutamicum* [90]. However, at concentrations of ½ MIC, the oxidized species were slightly less effective. This difference can be attributed to the lower hydrophobicity of the oxidized molecules, which affects their interaction with bacterial cell walls.

Moreover, membrane-active antibacterial terpenoids are less likely to cause resistance than small-molecule antibiotics. This was clearly demonstrated in the case of a CIT-derived synthetic polymer, citronellol-poly(*N*,*N*-dimethyl ethyl methacrylate), which was used to modify cotton fabrics [198]. The polymer exhibited high bactericidal activity against *E. coli*, *S. aureus*, and drug-resistant MRSA (e.g., MIC against *E. coli* was >31 µg mL^−1^, surpassing that of some commonly used biocides), due to the synergistic action of the membrane-active CIT and the positively charged PDMAEMA blocks. Despite the complex synthetic procedure and the need for an organic solvent to apply the hybrid polymer by soaking the fabric, the antimicrobial effect was worth pursuing. The antibacterial performance of the treated cotton fabric remained stable even after 40 washes (the antibacterial effect remained almost unchanged after 15 consecutive passages), and the polymer did not induce bacterial resistance.

### 4.4. Active Packaging Materials

**Figure 15:**
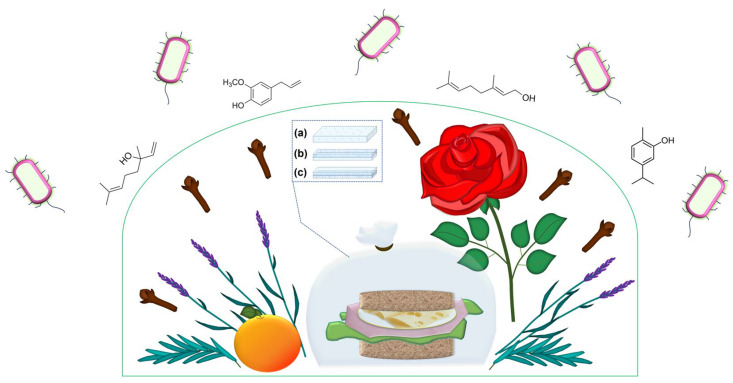
Natural essential oils as components of active packaging materials for food (inset: a–c—various structures of polymer films containing EOs).

The antioxidant and antibacterial properties of natural essential oils make them a popular choice as natural, renewable, and economical components in active food packaging materials (Figure 15) [199,200]. While essential oils are generally safe and non-cytotoxic at concentrations that affect pathogenic microorganisms [5,201], their overall effect depends on the essential oil’s composition [202,203]. Incorporating EOs into polymeric materials should also increase their safety level, protect them from direct interaction with food (e.g., proteins, carbohydrates, fats, salts, and changes in pH) [204], and improve the mechanical and oxidative stability and barrier properties of polymer matrices. For instance, binary essential oil mixtures containing thymol, carvacrol, citral, and eugenol, incorporated into thin films of polypropylene (PP)/polyamide (PA)/nanoclay composites, inhibited the growth of *E. coli* and fungi on sliced bread for over 50 days [205]. However, replacing petroleum-based polymers with macromolecules derived from renewable and/or waste materials can reduce the environmental impact of plastics [206,207,208]. Adding natural essential oils to such eco-friendly materials creates functional, active systems that can reduce both food loss and packaging waste.

#### 4.4.1. Synthetic Biocompatible Polymers

**Figure 16:**
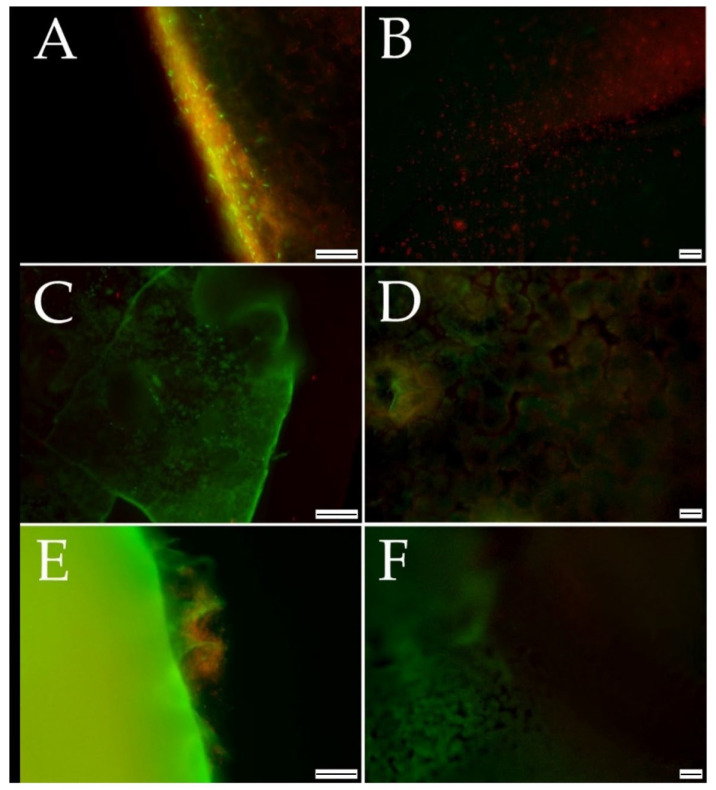
Fluorescence microscopy of *S. maltophilia* viability assay on polymers before and after modification with 3% *w*/*v* thymol (T): PLA (**A**); PLA/T (**B**); PBS (**C**); PBS/T (**D**); PBAT (**E**); PBAT/T (**F**) (insets: scale bar 20 μm) [211].

Biodegradable poly(butylene succinate) (PBS), blended with geraniol (8 wt.%), was processed into samples of two different microstructures (solid and porous) to form a material suitable for use as an insert in food containers and extended the shelf life of bread [209]. Migration tests confirmed that GER was slowly released from both the solid and porous plates over a period of more than 30 days, and under the applied conditions, the shelf life of bread increased by ~5 and 10 days (for the solid and porous samples, respectively). The PBS/GER blend was also used as an active coating to extend the shelf life of bread on paper primed with ethylene vinyl alcohol (EVOH) (the controlled release component) [210]. The multicomponent material effectively inhibited the growth of *B. cereus* and *E. coli* when the content of GER in the PBS matrix was 10 wt.%. Incorporation of thymol and eugenol in PLA, PBS, and poly(butylene adipate terephthalate) (PBAT) enhanced the antibacterial activity of the polymer matrices against *E. coli*, *S. aureus*, *Bacillus pumilus*, *Bacillus subtilis*, *Bacillus tequilensis*, and *Stenotrophomonas maltophilia* isolated from dairy products (Figure 16) [211].

Investigations on the morphology, crystallinity, and thermal properties of cast-extruded films made of polylactic acid/polybutylene succinate (PLA/PBS) blended with the lesser galangal (*Alpinia officinarum* Hance.) essential oil (LGEO; containing β-ocimene, 2-bornanone, eucalyptol, and geraniol) showed that the surface roughness decreased in the presence of LGEO [212]. Plasticization of the polymer blends with LGEO also modified the polymer crystals’ morphology, resulting in decreased crystallinity and a lower α-relaxation temperature. Tensile strength decreased by up to 22%, and water vapor and oxygen permeability increased by up to 42% and 29%, respectively, when the blends contained up to 6% LGEO. The blends were tested for their potential to delay mold and bacterial growth in steamed glutinous rice. The shelf life of cooked rice was extended by more than twofold, depending on the essential oil concentration and the PLA/PBS ratio. The films with lower contents of PLA exhibited greater efficacy in delaying fungal growth due to the more effective release of volatiles (LGEO plasticized PBS more effectively than PLA).

On the other hand, the barrier, mechanical, thermal, and antimicrobial properties of PLA could be improved by simultaneously reinforcing it with a poly(ethylene vinyl acetate) copolymer (GNP) and blending it with geraniol [213]. The properties of single-layered films (SLF) and multilayer films (MLF), produced by a coextrusion process (PLA-GER layer in the core), were compared. Although the thermal stability of the MLF was higher than that of SLF by ~8 °C, the water/gas barrier properties did not improve due to GNP agglomeration. Tensile measurements showed an improvement in mechanical rigidity of the blend (>87%). The MLF displayed high antimicrobial properties when used to package chicken samples for three weeks of storage under refrigerated conditions, protecting them against both G+ and G- pathogens.

#### 4.4.2. Biopolymers

Recently, studies on active food packaging have shifted towards edible polymeric materials. Pullulan (α-1,4-; α-1,6-glucan’) is an edible polysaccharide consisting of maltotriose units produced by the fungus *Aureobasidium pullulans* from starch. It has a variety of applications, including biomedical and pharmaceutical uses, such as drug delivery, the formation of medical devices, implant and tissue engineering, as well as in cosmetics and food packaging materials [214]. Emulsified pullulan films incorporating geraniol are transparent (> 90%), hydrophilic (water contact angle < 90°), and effective against *E. faecalis* ATCC 29212 and *P. aeruginosa* ATCC 27853 (with inhibition zone diameters of ~15 mm and ~11 mm, respectively) [215]. Pullulan loaded with GER also inhibited the growth of *E. faecalis* ATCC 29212 biofilms. The efficacy of starch coatings containing linalool, carvacrol, or thymol in protecting against *S. aureus* were studied in vitro and when inoculated onto the surface of Cheddar cheese [216]. The inhibitory effect of EOs increased significantly with their concentration in a solid medium. The inhibition of the growth of *S. aureus* on the cheese surface decreased in the following order: thymol > carvacrol > linalool.

**Figure 17:**
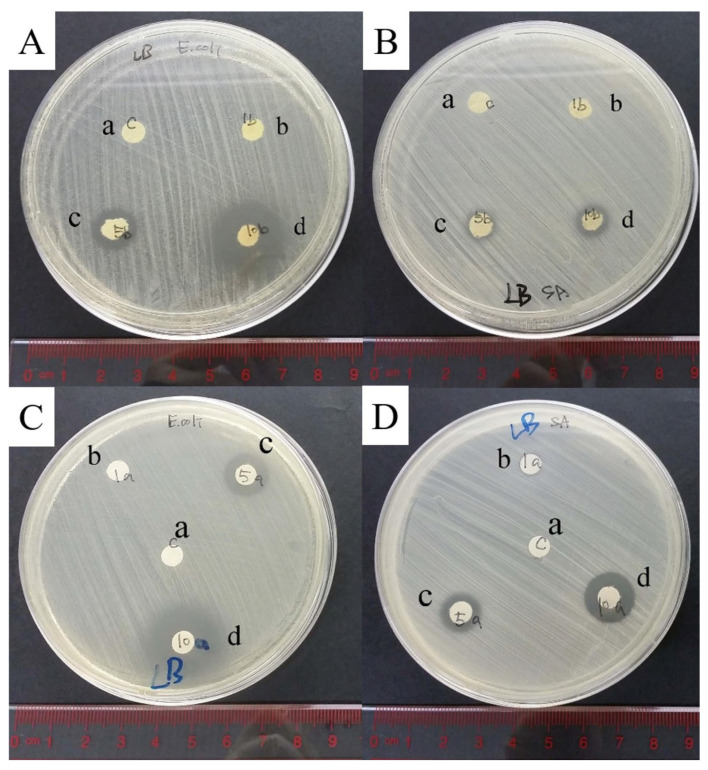
Bactericidal effect of EUG (*v*/*v*) added to films made by zein, PLA, and PEG: (a) ZPG_1:1_0.2 (a blend of zein and PLA with a mass ratio of 1:1 with a 20% PEG concentration), (b) ZPG_1%, (c) ZPG_5%, and (d) ZPG_10% against (**A**) *E. coli* and (**B**) *S. aureus*. Antimicrobial activity of eugenol on filter papers (FPs): (a) FP (no EUG), (b) FP_1%, (c) FP_5%, and (d) FP_10% against (**C**) *E. coli* and (**D**) *S. aureus*. [218]. Reprinted from Food Chem., 384/1, Huang, X.; Ge, X.; Zhou, L.; Wang, Y., Eugenol embedded zein and poly(lactic acid) film as active food packaging: Formation, characterization, and antimicrobial effects, 132482, Copyright (2022), with permission from Elsevier.

Eugenol, cinnamaldehyde, and carvacrol were added to zein-based materials (a corn-derived polymer) to enhance their antimicrobial properties against *S. aureus* and *E. coli* [217]. A gradual release of the essential oils (over a period of up to 96 h) was demonstrated with the strongest antimicrobial activity observed for cinnamaldehyde against *S. aureus*. CAR was only effective at a concentration of 3% *v*/*v*, for both bacteria. EUG showed no inhibitory effect on *S. aureus* and *E. coli* at concentrations of ≤ 3%. *E. coli* was only more sensitive than *S. aureus* at 8% *v*/*v* of EUG. Conversely, EUG significantly inhibited the growth of both *S. aureus* and *E. coli* in a blend of zein and PLA [218]. Figure 17 shows that the migration rate of EUG in various environments was controlled by zein.

Biodegradable and edible films and coatings made from gelatin and chitosan, with added eugenol and oregano (*Origanum vulgare*) essential oil (OEO) were shown to be effective against *S. aureus* and *E. coli* [219]. The effect was attributed to the synergistic antimicrobial activity of EUG, OEO, and chitosan. Chitosan-based composite coatings (admixed with gelatin, starch, and sorbitol and modified by the addition of geraniol or thymol) were designed as active, biodegradable postharvest protective coatings against *Botrytis cinerea* fungi on fresh strawberries [220]. The coated fruits were inoculated with fungal spores and stored for 7 days at 4 °C. Although the chitosan coatings were already effective without EOs, a significant improvement was achieved with the coating comprising 1% chitosan, 1% starch, 0.5% sorbitol, 0.05% tween, and 0.02% phenolic thymol. It has been also reported that strawberries treated with EUG grafted onto carboxymethyl cellulose modified with cysteamine hydrochloride can be stored for 7 days [221]. Furthermore, the shelf life of strawberries could be extended by countering microbial deterioration with edible coatings containing onion waste extracts (with components such as β-sitosterol, phytol, 1-butanol, 3-methylformate, and 5-hydroxymethylfurfural) [222].

Both pectin (a heteropolysaccharide derived from plants and built of D-galacturonic acid) and collagen (a protein found in animal connective tissue) are structural polymers that are often used as gelling agents [223]. They are compatible with each other, and a combination of pectin and collagen was used in composite hydrogel-based films enriched with essential oils derived from *Melissa officinalis* (a mixture of 2,6-octadienal, 3,7-dimethyl, citral, caryophyllene, geranyl acetate, caryophyllene oxide, citronellal, and linalool) at varying concentrations (0.1%, 0.15%, and 0.2%) [224]. FTIR analysis confirmed interactions between the EOs components and the polymer matrix. The film transparency decreased by increasing the content of the phytochemicals, and the most homogeneous structure was obtained by adding 0.2% EOs. The elongation at break of the hybrid films increased, but their tensile strength decreased. The films also demonstrated improved antioxidant and barrier properties against water vapour.

Kefiran (an exopolysaccharide produced by *Lactobacillus* species [225]) and calcium caseinate (calcium salt of a natural protein polymer found in milk [226]) can be used to produce films and nanofibers for active food packaging applications. Films based on kefiran/gelatin [227] and gelatin/calcium caseinate [228] were admixed with phytochemicals in the form of *Zhumeria majdae* essential oil nanoemulsion (ZMEO-NE) and petitgrain (*Citrus aurantium*) essential oil (PEO), respectively. The main components of *Z. majdae* EOs were linalool and camphor, whereas the petitgrain oil contained linalyl acetate, linalool, α-pinene, α-terpineol, limonene, β-myrcene, geranyl acetate, and neryl acetate. Adding ZMEO-NE to the kefiran/gelatin films (8% *v*/*v*) decreased their water vapor permeability by almost twofold, as well as their glass transition temperature, storage modulus, tensile strength (from 38.44 to 33.48 MPa), and light transmission but increased their elongation at break (from ~20 to ~35%). EOs also caused changes in the surface morphology and crystalline structure of the film. These effects can be attributed to the formation of hydrogen bonds between the EOs and the polymer network. Increasing the ZMEO-NE concentration also enhanced the antioxidant potential of the kefiran/gelatin films and their antibacterial efficacy against *S. aureus* and *E. coli*. FTIR confirmed the presence of intermolecular interactions between gelatin/calcium caseinate and PEO. The antioxidant capacity of gelatin/calcium casein films, their thickness, thermal stability, elongation at break, and water vapour permeability (and also water solubility), increased significantly upon increasing the PEO concentration. The active films showed strong antimicrobial activity against *C. albicans* and *P. aeruginosa*. However, after adding essential oils, the films became yellowish, and their transparency, moisture content, and tensile strength decreased.

## 5. Conclusions and Future Perspectives

This article presents the current state of knowledge in the design of polymeric materials with antimicrobial and antibiofilm properties based on the use of phytochemicals—components of essential oils. Terpenes, terpenoids, and phenolic compounds present in mixtures of secondary metabolites synthesized by various plants are valuable therapeutic agents, known for their medicinal uses and for combating pathogens by blocking communication mechanisms that lead to increased virulence, especially in drug-resistant bacterial strains. Recently, extensive microbiological and metabolomic studies have been conducted to elucidate the interactions underlying the mechanism of antimicrobial action of essential oils. The results of these studies, combined with *in silico* analyses of supramolecular interactions between phytochemicals and various amino acids in microbial proteins and enzymes, not only explain the nature of antimicrobial activity of these compounds but also provide a solid basis for designing new polymer systems in which active compounds are not only blended with macromolecules but also incorporated by covalent grafting. In this way, the activity of essential oils can be preserved or at least only slightly reduced when they are, for example, permanently bound to the surfaces of polymer coatings. This approach not only preserves the volatile components of essential oils but also targets their antimicrobial activity, increasing their safety in clinical settings.

The prospects for the development of antibacterial macromolecular systems based on phytochemicals in counteracting the development of bacterial resistance are promising. The use of macromolecular nanocarriers (nanocapsules, nanofibers, and inclusion complexes) can improve the stability, bioavailability, and cellular uptake/internalization of these natural compounds, as well as reduce their toxicity. These systems can be used for various drug delivery methods in biomedical applications, including skin treatment and wound healing. Essential oils can also be used effectively in materials intended for active packaging applications. Although information on the synergistic therapeutic effects of essential oils in combination with other bioactive substances is available in studies on pure compounds, further research is needed to fully harness these specific properties in polymer systems.

## Figures and Tables

**Table 1:** Summary of the antimicrobial activity of selected phytochemicals.

Name	Type *	Advantages	Ref.	Disadvantages	Ref.
Eugenol	A	Active against both G+ and G- bacteria.	[38,44,45,46]	Masking phenolic group changes antimicrobial activity of EUG.	[38,44]
		Eradication of planktonic cells of *S. epidermidis* and *P. aeruginosa*.	[39]	Less active against biofilms of *S. epidermidis* and *P. aeruginosa*.	[39]
		Hinders quorum sensing in *E. coli* and *S. aureus*.	[48,54]		
		Affects virulence and NorA efflux pump in *S. aureus*.	[49,52]		
		Affects MexA and AcrA efflux pumps in *P. aeruginosa* and *E. coli*.	[53]		
Carvacrol	A	Active against both G+ and G- bacteria.	[38,44,45,46]	Less active against biofilms of *S. epidermidis* and *P. aeruginosa*.	[39]
		Eradication of planktonic cells of *S. epidermidis* and *P. aeruginosa*.	[39]	Weaker effect on *P. aeruginosa* biofilms at sublethal concentrations.	[61]
		Eradication of KPC-producing *K. pneumoniae*.	[59]		
		Antibiofilm and antivirulence action against UPEC *E. coli*.	[60]		
		*S. aureus* biofilm inhibition.	[61]		
		Inhibiting/modulating biofilm formation in *P. aeruginosa*	[62]		
		amplification of the action of antibiotics.	[64]		
Linalool	B	Cell membrane destruction and alteration of bacteria metabolism.	[68]		
		Inhibition of *P. aeruginosa, A. hydrophila*, and *S. aureus* bacterial growth.	[69,70,71,75,76]		
		Eradication of *E. coli*.	[73]		
		Hinders quorum sensing in *A. baumannii* and *P. aeruginosa*.	[72,74]		
		Inhibition of *B. amyloliquefaciens* motility.	[77]		
Geraniol	B	Antibiotic activity against *Streptococcus* spp., *Staphylococcus* spp., and *S. aureus* (MRSA).	[80,81,82,83,84]	Less active against *Staphylococci* than citral and LIN.	[66]
		Effective (almost as citral) against *Enterococci*.	[66]		
		Bactericidal activity against *E. coli* and *H. pylori*.	[85,86]		
		Inhibition of *A. baumannii*, *S. epidermidis*, *E. carotovora*. and *P. fluorescens* biofilm formation.	[55,81,87]		
Citronellol	B	Antimicrobial and antibiofilm action towards *E. coli*.	[89]		
		Antimicrobial action against *E. coli*, *S. aureus*, and *Corynebacterium glutamicum* bacterial strains.	[90]		
		Inhibition of NorA efflux pump in *S. aureus*.	[91]		
Farnesol	C	Quorum-sensing molecule in *Candida albicans* biofilm (reactive oxygen species production, induction of cell apoptosis, and modulation of virulence factors).	[94]	Only *E,E*-isomer is active against *C. albicans*.	[95]
		Sensitized *S. aureus* towards antibiotics (gentamycin and β-lactam antibiotic).	[96,97]	*E,E*-isomer acted only on G+ bacteria.	[96,97,98]
		Ethanolic solutions of FAR-inhibited biofilms of *S. aureus*, *P. aeruginosa*, and *S. epidermis*.	[99,100]	Enhanced tolerance of *S. aureus* to some antimicrobials.	[101]
Phytol	D	Increased the level of ROS in *P. aeruginosa*.	[109]	Antagonistic interactions between PHY and norfloxacin in *S. aureus*.	[110]
		Enhanced effects of norfloxacin against *Salmonella* spp. and *E. coli*.	[110,111]		
		*K. pneumoniae* biofilm eradication.	[113]		
		Inhibition of quorum sensing in *P. fluorescens*, *P. aeruginosa, C. violaceum*, *Serratia marcescens*, *L. monocytogenes*, *S. enterica*, and *E. coli*.	[114,116,117,118]		
		Eradication of *A. baumannii* biofilm in combination with cefotaxime.	[115]		

* A—monoterpenoid phenol; B—acyclic monoterpenoid alcohol; C—acyclic sesquiterpene alcohol; D—hydrogenated diterpene alcohol.

## Data Availability

No new data were created or analyzed in this study. Data sharing is not applicable to this article.

## Abbreviations

The following abbreviations are used in this manuscript:

EOs	Essential oils
G+	Gram positive
G-	Gram negative
EPS	Extracellular polymeric substances
QS	Quorum sensing
QQ	Quorum quenching
AHL	Acylated homoserine lactone
EP	Efflux pumps
MIC	Minimum inhibitory concentration
MBC	Minimum bactericidal concentration
MRSA	Methicilin-resistant Staphylococcus aureus
VRSA	Vanomycin-resistant Staphylococcus aureus
EUG	Eugenol
GAR	Carvacrol
LIN	Linalool
GER	Geraniol
CIT	Citronellol
FAR	Farnesol
PHY	Phytol
LNCs	Lipid nanocapsules
CD	Cyclodextrin
IC	Inclusion complex
NF	Nanofiber
NP	Nanoparticle
